# Biodegradable Zn‐Based Implants: Progress, Challenges, and Pathways toward Clinical Translation

**DOI:** 10.1002/advs.76066

**Published:** 2026-06-15

**Authors:** Panfeng Zhao, Zhengquan Wang, Hui Yang, Xiyuan Zhang, Hanyuan Liu, Xiaotong Lu, Xiaocheng Li, Yanyan Liu, Xi Zhao, Wenhao Zhou, Yufeng Zheng

**Affiliations:** ^1^ School of Materials Science and Engineering Northeastern University Shenyang China; ^2^ Shaanxi Key Laboratory of Biomedical Metallic Materials Northwest Institute for Non‐Ferrous Metal Research Xi'an China; ^3^ School of Materials Science and Engineering Peking University Beijing China

**Keywords:** antibacterial activity, biocompatibility, clinical translation, corrosion control, Zn‐based biodegradable implants

## Abstract

As a new generation of biomaterials, biodegradable zinc (Zn)‐based implants hold substantial promise for clinical use due to their favorable biocompatibility and controllable degradation behavior. This review summarizes recent progress in Zn‐based implants, focusing on the physiological roles of Zn, current Zn‐based implant systems, and relevant applicable standards. To address the critical challenges faced in practical applications, such as optimizing corrosion rate, antibacterial performance, and biocompatibility, researchers have employed innovative strategies, including alloying (e.g., Zn‐Mg, Zn‐Mn, Zn‐Li), surface modifications, and functional coatings. Meanwhile, a detailed overview is provided of in vitro and in vivo degradation evaluation systems, advances in preclinical animal studies, and the status of clinical trials, together with regulatory requirements for biodegradable metals set by domestic and international authorities. Key mechanistic and translational bottlenecks that currently limit clinical adoption are identified, together with critical directions for the rational design and standardized evaluation of next‐generation Zn‐based implants.

## Graphical abstract

Exploring biodegradable Zn‐based implants offers a promising pathway to next‐generation biomedical devices with balanced degradation and biocompatibility. A comprehensive overview of biodegradable Zn‐based implants, covering their biological significance, material design principles, and advanced engineering strategies is provided. Key challenges, including corrosion control, antibacterial performance, and biocompatibility, are critically discussed alongside recent progress in alloy development and surface modification. evaluation methodologies, preclinical and clinical advancements, and regulatory considerations, highlighting future pathways toward clinical translation are also further outlined.

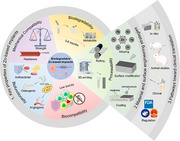

**Abbreviations**
3D: Three‐dimensional
ABL: Abaloparatide
AD: Anodizing
Ag: Silver
Al: Aluminium
ALP: Alkaline Phosphatase
AM: Additive Manufacturing
ASA: Acetylsalicylic Acid
ASTM: American Society for Testing and Materials
BCWC: Bottom Circulating Water‐cooled Casting
BIC: Bone‐implant Contact
BMD: Bone Mineral Density
BMP: Bone Morphogenetic Protein
BSP: Bone Sialoprotein
Ca: Calcium
Ca_3_(PO_4_)_2_
: Calcium Phosphate Tribasic
CE: European Conformity
CR: Corrosion Rate
CS: Chitosan
c‐SBF: conventional simulated body fluid
Cu: Copper
DNA: Deoxyribonucleic Acid
DRX: Dynamic Recrystallization
E: Elongation
*E. coli*
: *Escherichia coli*
EBSD: Electron Backscattered Diffraction
ECAP: Equal Channel Angular Pressing
EDS: Energy Dispersive Spectroscopy
EIS: Electrochemical Impedance Spectroscopy
EMA: European Medicines Agency
EP: Electrplishing
EPD: Electrophoresis Deposition
FDA: Food and Drug Administration
Fe: Iron
GO: Graphene Oxide
H_2_O: Water
HA: Hydroxyapatite
HE: Hematoxylin‐eosin
HPT: High‐pressure Torsion
HR: Hot Rolling
HT: Heat Treatment
IAI: Implant‐associated Infections
ICH: the International Council for Harmonisation of Technical Requirements for Pharmaceuticals for Human Use
IFN‐γ: Interferon‐gamma
IL‐1β: Interleukin‐1beta
ISO/Ts: International Organization for Standardization/Technology Specification
KFDA: Korea Food and Drug Administration
Li: Lithium
L‐PBF: Laser—Powder Bed Fusion
MAO: Microarc Oxidation
MDR: Medical Device Regulation
Mg: Magnesium
MHC: Major Histocompatibility Complex
Micro‐CT: Micro Computed Tomography
Mn: Manganese
MTs: Metallothioneins
NF‐κB: Nuclear Factor Kappa‐B
NMPA: National Medical Products Administration
O_2_
: Oxygen
OCN: Osteocalcin
OCP: Open Circuit Potential
OPN: Osteopontin
PBS: Phosphate Buffered Saline
PDA: Polydopamine
PDE: Phosphodiesterase
PEO: Plasma Electrolytic Oxidation
pH: Potential of hydrogen
PTP: Protein Tyrosine Phosphatase
ROS: Reactive Oxygen Species
r‐SBF: revised simulated body fluid
*S.aureus*
: *Staphylococcus aureus*
Sc: Scandium
SEM: Scanning Electron Microscope
SIM: Simvastatin
SPD: Severe Plastic Deformation
Sr: Strontium
Th: T Helper Cell
Ti: Titanium
TLCS: Total Local Citation Score
TMP: Thermomechanical Processing
TNF‐α: Tumor Necrosis Factor‐alpha
UTS: Ultimate Tensile Strength
YS: Yield Strength
ZIP: Zinc‐regulated Transporters, Iron‐regulated Transporter‐Like Protein
Zn: Zinc
Zn(OH)_2_
: Zinc Hydroxide
Zn_3_(PO_4_)_2_
: Zinc Phosphate
ZnO: Zinc Oxide
ZnT: Zinc Transporter
ZrO_2_
: Zirconium Oxide

## 1 Introduction

Implantable devices play a crucial role in modern medicine, serving as indispensable tools for treating conditions such as fractures, bone defects, and cardiovascular diseases [1]. Conventional metal implants, such as stainless steel, titanium alloys, and cobalt‐chromium alloys [2], have been widely used in clinical practice for their excellent mechanical properties and long‐term clinically proven stability [3]. However, keeping these implants in the body for long periods may lead to a range of complications [4], such as stress shielding‐induced osteoporosis, inflammatory responses triggered by the release of metal ions, and the pain and risks associated with secondary removal surgery [5]. With the interdisciplinary development of materials science and medicine, biodegradable biomaterials have emerged as an important research direction. These materials provide temporary mechanical support while gradually degrading and being absorbed after tissue repair, offering new insights and development opportunities for the design of next‐generation implants [6].

**FIGURE 1:**
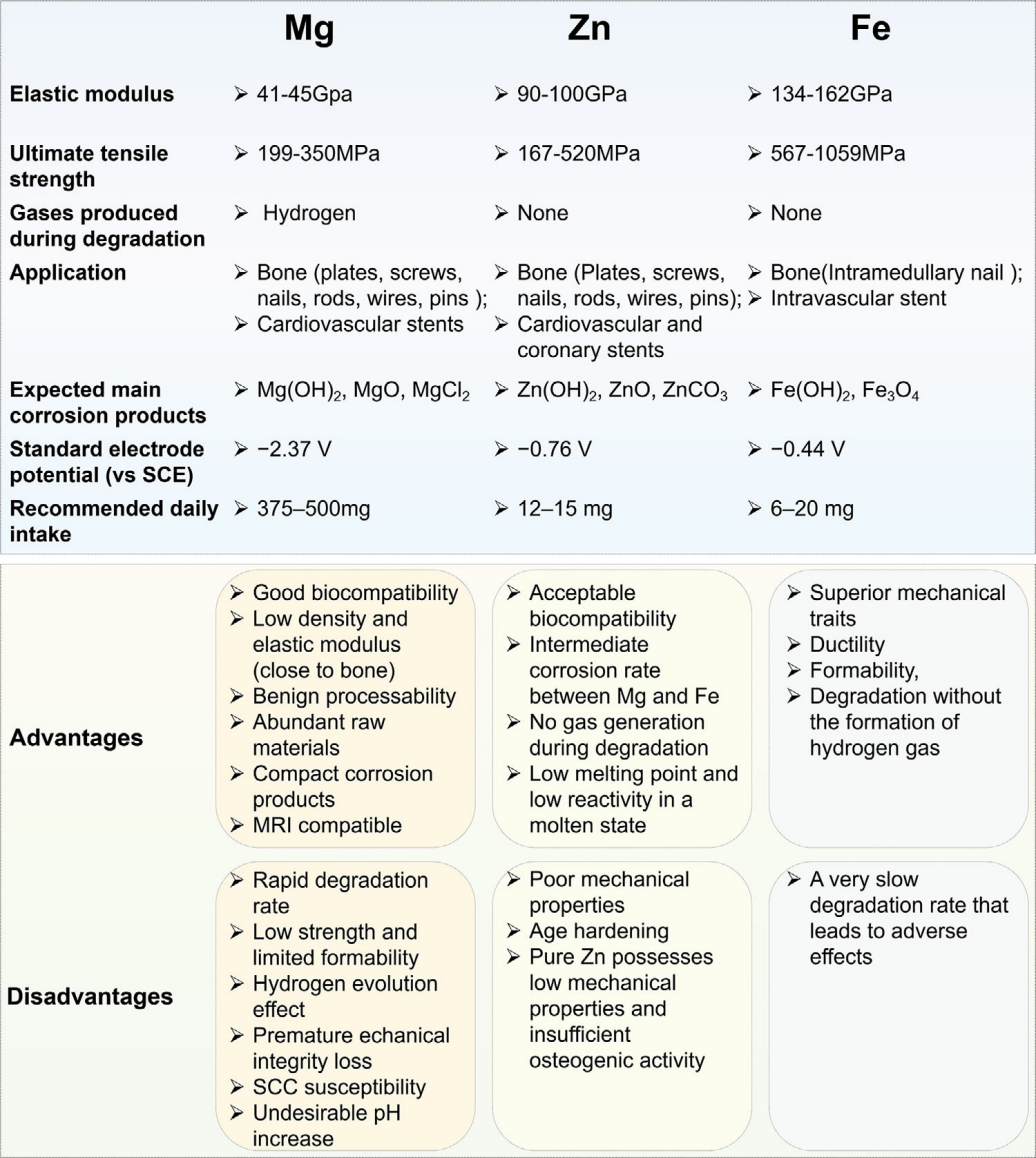
Comparison of biodegradable metals based on Mg, Zn, and Fe [11, 12].

**FIGURE 2:**
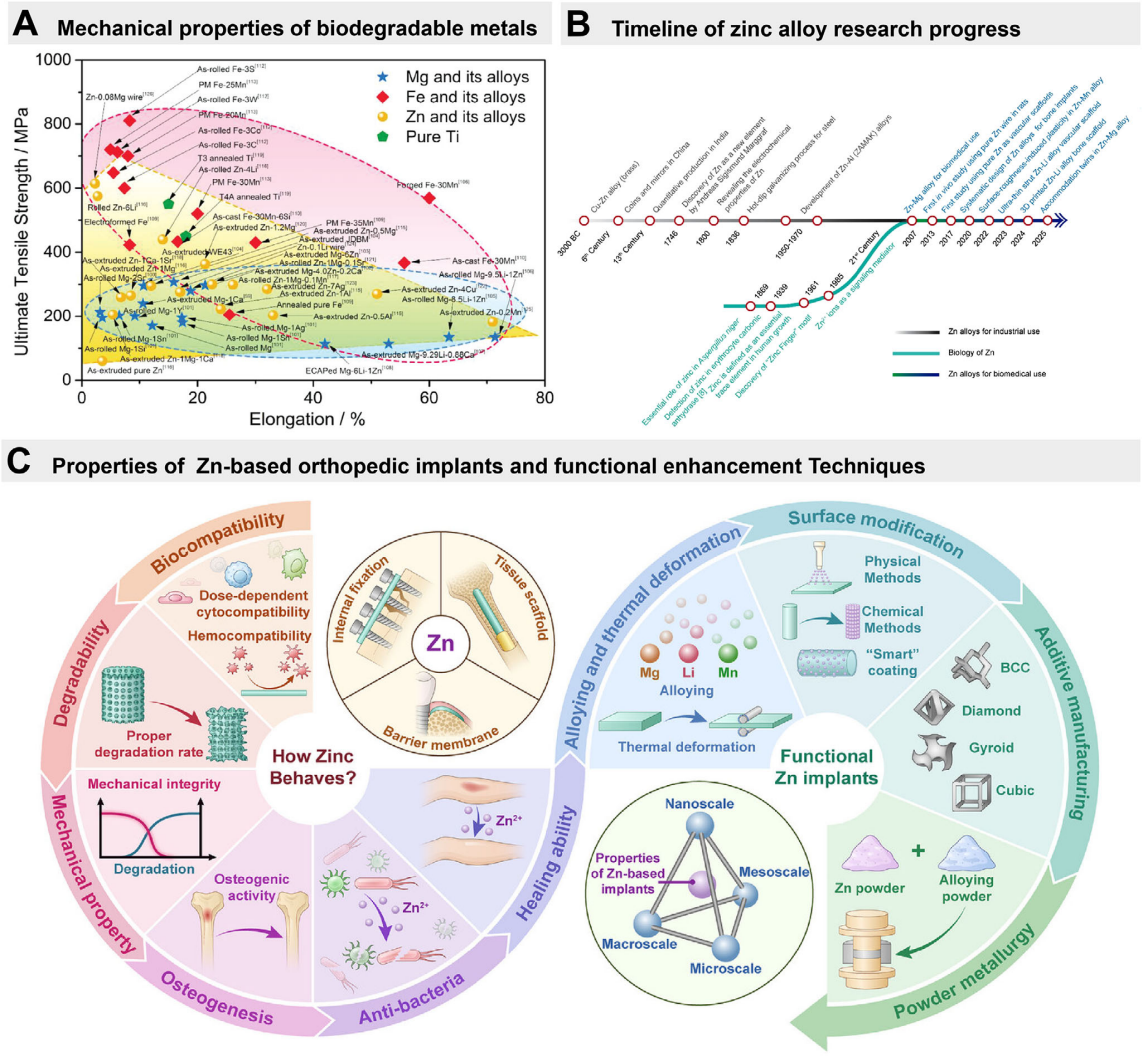
Mechanical behavior, historical progress and biological interactions of Zn metal and Zn‐based implants. (A) Illustration of the tensile behavior of Mg‐, Fe‐, and Zn‐based biodegradable metals in air at room temperature [14]. Copyright 2019, John Wiley and Sons. (B) Timeline of key advances in understanding the material and biological properties of Zn [25].Copyright 2025, Elsevier. (C) Material properties and biological features of biodegradable Zn‐based orthopedic implants, together with current engineering strategies for tuning the properties of functionalized Zn‐based systems across multiple scales [11]. Copyright 2025, John Wiley and Sons.

Among biodegradable metals, Mg and Fe were the first to receive broad attention. Mg metal shows good biocompatibility and an elastic modulus close to bone, but its rapid corrosion and degradation rates often lead to early loss of mechanical support and may cause local hydrogen release [7]and changes in the alkaline environment [8]. Fe metal has good mechanical strength but degrades too slowly to match bone healing [7, 9], and long‐term retention may trigger tissue responses [5]. Given these limitations, Zn has recently gained interest as a promising alternative. Zn naturally participates in key physiological activities, such as signal regulation, gene expression and nucleic‐acid metabolism [10]. Its degradation rate is moderate, faster than Fe but slower than Mg [7]. From a biocompatibility perspective, Zn^2+^ participates in various biochemical reactions, shows low cytotoxicity and can promote cell growth and differentiation. Its overall compatibility with bone exceeds that of stainless steel and titanium alloys [11, 12, 13] (Figure 1). Zn‐based implants hold promise in maintaining adequate mechanical support while better matching the bone tissue repair process (Figure 2A) [14, 15]. Beyond orthopedics, Zn metal has also attracted interest in cardiovascular applications [16, 17, 18]. Zn‐based biodegradable stents show suitable degradation behavior and strong biocompatibility in blood vessels, reducing chronic inflammation and restenosis linked to long‐term use of permanent metal stents [19, 20, 21, 22]. Simultaneously, Zn^2+^ helps regulate endothelial cell growth and smooth‐muscle cell migration, supporting vascular repair and remodeling [23, 24].

Based on the above reasons, significant progress has been achieved in studies of Zn‐based implants both domestically and internationally since the inception of research on biodegradable metallic implants [11, 25]. A recent review on biodegradable Zn summarizes the development of Zn alloys from industrial applications to their current use in biomedical applications (Figure 2B) [25]. Furthermore, researchers have conducted extensive work on the alloy design, surface modification, and the assessment of their biological behavior in *vitro* and in *vivo*, aiming to further optimize their properties and promote their better application in clinical settings (Figure 2C) [11]. While these studies have summarized the development of Zn alloys, most have focused on component optimization and surface modification, with limited evaluation of clinical applications and implant regulation. This paper systematically summarizes the current advancements in Zn‐based implant research. Based on existing studies, it provides a comprehensive overview of the latest international standards governing biodegradable metallic implants and the corresponding clinical‐oriented design principles for implantable materials, including degradation behavior, antibacterial performance, and biocompatibility. Moreover, mainstream strategies for improving the performance of Zn‐based alloys, such as alloying, surface modification, and functional coating, are comparatively analyzed. In addition, the present progress, limitations and core bottlenecks of preclinical animal studies and clinical trials supporting the clinical translation of Zn‐based implants are comprehensively sorted out, covering the whole process from material design to clinical application. We also proposed emerging areas for clinical applications of biodegradable Zn implants. The summary of the above content provides crucial support for researchers studying Zn‐based implants, advancing their development within the field of biodegradable metallic implants.

**FIGURE 3:**
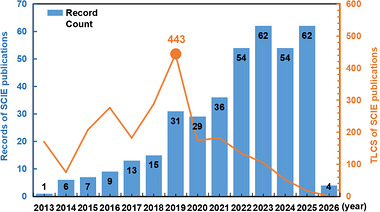
Global publication trends of Zn‐based biodegradable implants.

Methodologically, a statistical analysis is also performed to track yearly changes in SCIE‐indexed publications and the Total Local Citation Score (TLCS) on Zn‐based implants (Figure 3). The dataset was obtained from the SCI‐EXPANDED database in the Web of Science Core Collection. Publications were identified using the following search strategy: (Zn‐based implant*) (All Fields) OR (Zinc‐based implant*) (All Fields) AND bioabsorba* or biodegrada* (All Fields). A total of 383 articles were obtained, covering the period from 2013, the earliest record, to November 13, 2025 (Figure 3). In 2013, Professor Jaroslaw W. Drelich and colleagues at Michigan Technological University first proposed zinc as a promising biodegradable implant material, particularly for vascular stents [22].They reported that zinc exhibits a moderate degradation rate (approximately 20–200 µm/year), lying between that of magnesium (too rapid) and iron (too slow). Moreover, released Zn^2+^ ions were found to promote osteogenesis and possess antibacterial activity while posing minimal systemic toxicity. This pioneering discovery established the foundation for subsequent research on Zn‐based implants.

## 2 Overview of Zn‐Based Implant Systems

### 2.1 Biological Significance of Zn

**FIGURE 4:**
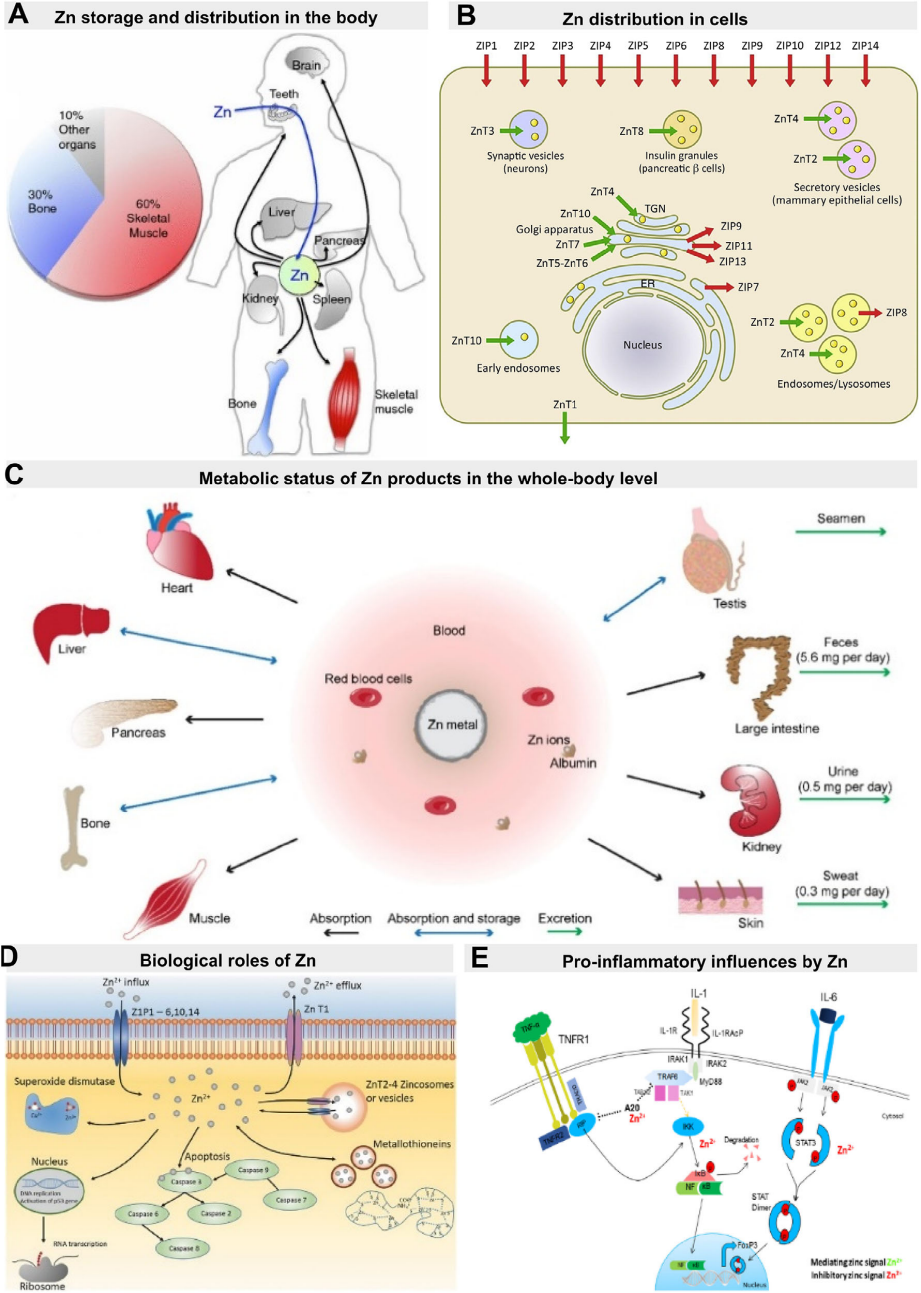
Multilevel Zn biology: systemic distribution, cellular transport, signaling pathways and physiological functions. (A) Zn storage and distribution in the body [27]. Copyright 2017, Elsevier. (B) Cellular Zn distribution [29]. Copyright 2019, Elsevier. (C) Systemic Zn transport, absorption, storage, and excretion [25]. Copyright 2026, Elsevier. (D) Zn influence on pro‐inflammatory signaling [30]. Copyright 2017, MDPI. (E) Biological roles of Zn [16]. Copyright 2016, John Wiley and Sons.

Zn is an essential trace element found throughout the human body [26]. Approximately 85% of Zn is stored in bones and muscles, 11% in the liver and skin, with the remainder distributed across other tissues (Figure 4A) [27]. The total Zn content in a normal adult human body is approximately 2 to 3 grams, with a recommended daily intake ranging from 5 to 20 mg [28]. Figure 4B,C illustrates the distribution and metabolism of Zn in vivo and in cells [25, 29].

### 2.2 Development and Current Status of Zn‐Based Implants

**FIGURE 5:**
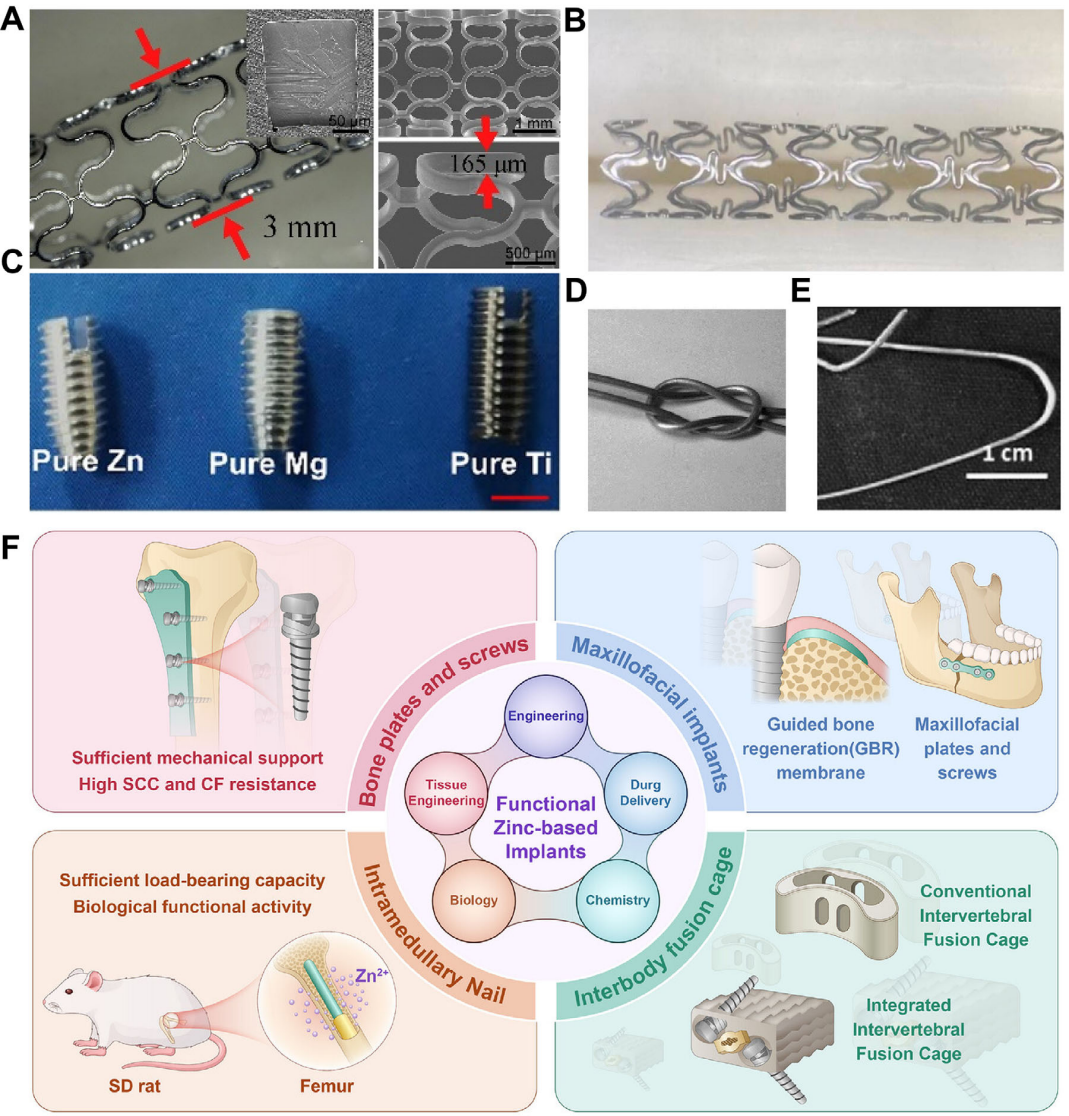
Representative Zn‐based implants and applications. (A) Optical and SEM images of Zn stents [32]. Copyright 2017, Elsevier. (B) Optical images of Zn‐Cu‐Mn stent [33]. Copyright 2025, Elsevier (C) Pure Zn, Mg, Ti screws [34]. Copyright 2021, Elsevier. (D) Tied surgical knot used in mechanical tests (Zn‐0.008Mg) [35]. Copyright 2021, Elsevier (E) Zn–Cu wires after 0 week of immersion testing [31]. Copyright 2023, Elsevier. (F) Clinical application potential of Zn‐based implants [11]. Copyright 2025, John Wiley and Sons.

As illustrated in Figure 5A–E [31, 32, 33, 34, 35], due to their unique properties, Zn‐based alloys have been used in the fabrication of implantable devices such as interface screws, vascular stents, suspension loop plates, gastrointestinal anastomosis staples, and rib plates [36, 37, 38]. Through rational alloying and processing techniques, Zn alloys can meet the requirements for fracture resistance and load‐bearing during screw implantation. Moreover, Zn alloys can promote bone healing through specific mechanisms, such as Zn‐Mn‐Mg promoting tendon‐bone integration [39], Zn‐Ag providing both antibacterial and anti‐osteolytic effects [40], and Zn‐Li activating osteogenesis‐related signaling pathways [41]. Additionally, biodegradable Zn‐based alloy stents have emerged as another significant area of research, driven by the goal of addressing the long‐term issues associated with traditional permanent stents, including inflammation, thrombosis, and restricted vascular function. Qian et al. [33] implanted Zn‐Cu‐Mn stents in pig coronary arteries for 18 months, pure Zn stents in rabbit abdominal aortas for 12 months [32], and in mouse arteries for 20 months [42]. Additionally, the Zn‐1.5Cu‐1.5Ag alloy developed by Chen [43], along with related in *vitro* and animal experiments, demonstrated that Zn‐based alloy stents exhibit excellent safety and biocompatibility. Li et al. [44] developed a novel biodegradable Zn‐0.8Cu‐0.03B alloy. Following in *vitro* validation, the alloy was implanted into the rabbit bile duct. The alloy stent maintained its structural integrity within the common bile duct for 3 weeks and achieved complete degradation within 8 weeks. The degradation products were metabolized safely without significant organ accumulation.

In addition, Zn‐Li‐Mn alloy staples [45], Zn‐Cu wire [31], and Zn‐Mg wire [35] have been used as implants such as sternum sutures and gastrointestinal anastomosis staples. These materials not only achieve tissue fixation and promote wound healing but also help avoid complications such as infections, foreign body reactions, and metal displacement (Figure 5F) [11].

### 2.3 Key Focuses of Zn‐Based Implant Research

**FIGURE 6:**
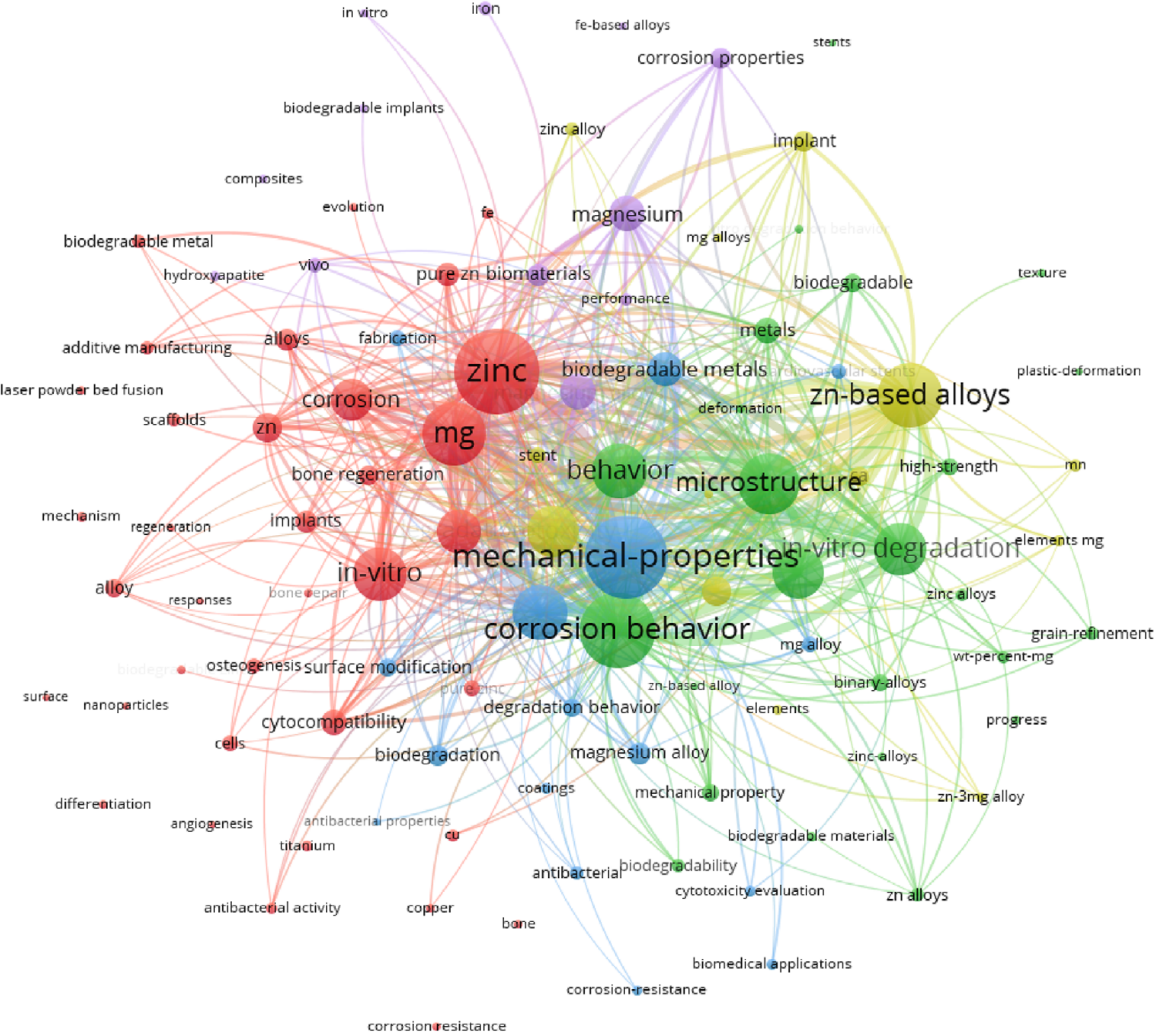
Key themes in Zn‐based implant research. The co‐occurrence network was built from 101 author keywords appearing more than eight times. Node size reflects keyword frequency, and nodes within the same thematic group share a color. Line density represents co‐occurrence strength between keyword pairs. Some labels were removed to maintain clarity.

To provide a visual analysis of the main concerns in Zn‐based implant studies, a program VOSviewer (version 1.6.8) was utilized for keyword network analysis based on the aforementioned bibliometric data. The VOSviewer time slicing was set from 1900 to 2025, and the keyword analysis was performed under the “co‐authorship” and “citation” modes, using “all keywords” as the analytical unit. As shown in Figure 6, a co‐occurrence network was constructed from the 101 author keywords that appeared more than eight times. The network shows that mechanical performance and corrosion behavior lie at its center, underscoring that clinically useful Zn‐based implants must retain load‐bearing ability during degradation while keeping their breakdown products biologically safe. The dense links around these core nodes indicate that microstructural control, alloying and surface modification now form the main design strategies.

In biocompatibility and functionalization, the field has moved beyond simple cytotoxicity tests and is beginning to use the biological roles of Zn^2+^ to enhance osteogenesis, angiogenesis and antibacterial effects. As a result, the assessment of Zn‐based implants should follow an integrated framework that joins material performance, biological outcomes and clinical considerations. Such a framework needs to include multidimensional checks of degradation behavior and tissue responses in both in *vitro* and in *vivo* settings, and it must also meet regulatory expectations and long‐term safety needs.

### 2.4 Standards and Regulatory Specifications for Zn‐Based Implants

As biodegradable Zn‐based implants progress from laboratory research toward clinical use, a clear and unified technical standard system is essential to ensure safety, effectiveness and quality control. Existing standards for stainless steel, titanium alloys and polymeric implants are not fully suitable for degradable metals. At present, the International Organization for Standardization (ISO) and the American Society for Testing and Materials (ASTM) have issued several guidelines specifically addressing biodegradable metallic materials.

ISO/TS 20721:2025 Surgical Implants – Absorbable Implants – General Guidelines and Requirements for the Evaluation of Absorbable Metal Implants, published in May 2025, replaces the 2020 edition [46]. This update reflects the properties of new absorbable metals, including Zn‐based alloys, by revising referenced documents and key technical parameters. Major improvements include tighter control of material composition, such as defined limits for alloying elements (e.g., Mg, Li, Cu) and impurities (e.g., Fe < 0.005%). The standard also requires assessment of microstructural effects on degradation uniformity to prevent early mechanical failure caused by local micro‐galvanic corrosion. A central concept introduced in this guideline is that Zn‐based implants must align their degradation rate with the bone‐healing timeline. According to ASTM F3268 testing recommendations [47], the in vitro degradation rate of Zn‐based materials in SBF should fall within 0.1‐0.5 mm/year, with local pH kept between 7.0 and 7.6 and hydrogen release below 0.5 mL/(cm^2^·d) to avoid inflammatory reactions or gas‐pocket formation.

In addition to ISO/TS 20721:2025, the ISO 10993 series [48, 49]remains the key international framework for biological safety evaluation, covering cytotoxicity, sensitization, irritation, systemic toxicity and genotoxicity. The regulatory system for degradable Zn‐based implants continues to evolve. As more clinical evidence becomes available, the standards will likely be refined and expanded to include product‐specific requirements, such as those for bone screws.

## 3 Design Principles of Zn‐Based Implants

**FIGURE 7:**
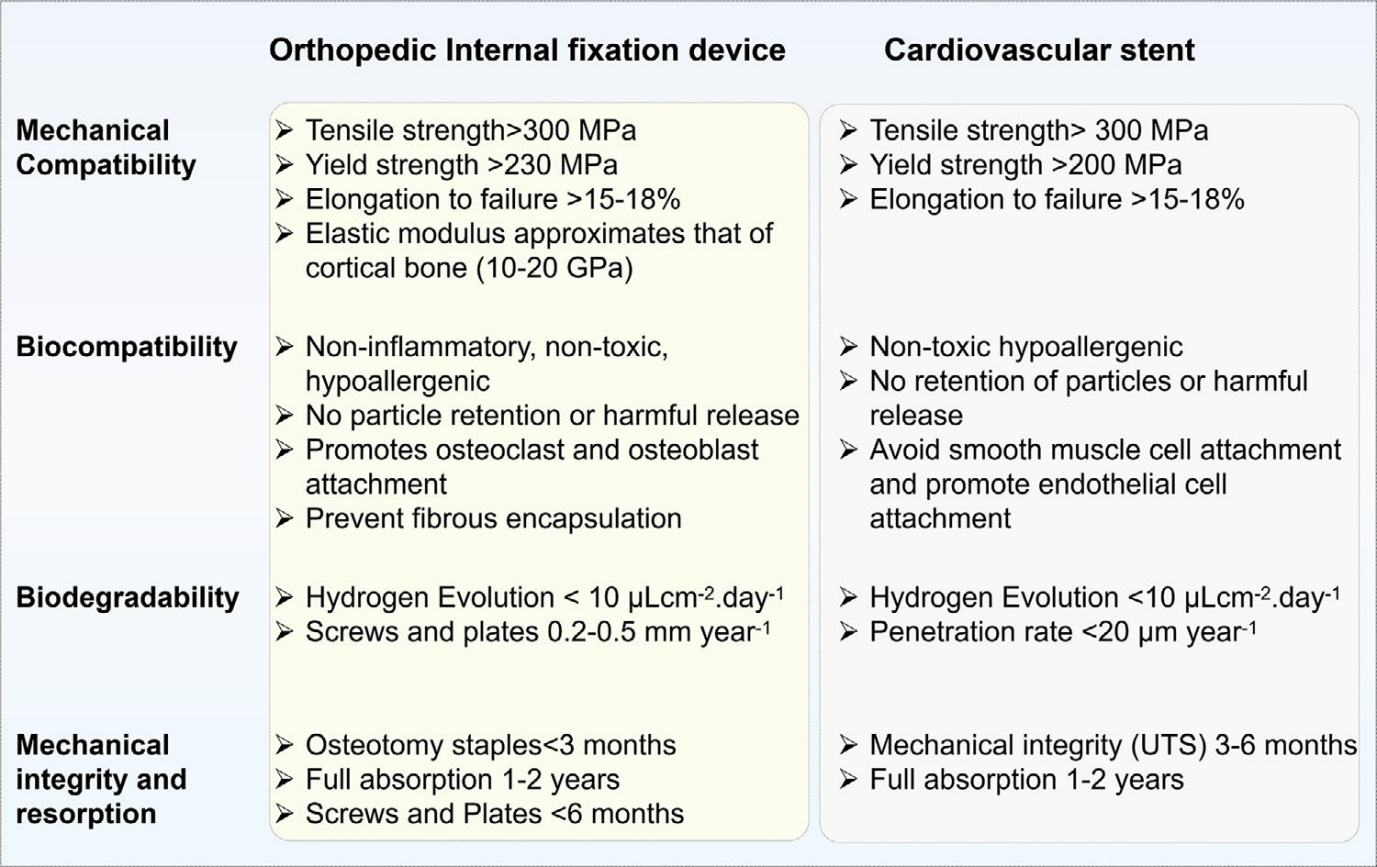
Key design criteria for biodegradable metallic implant devices in cardiovascular and orthopedic applications [13].

To ensure the clinical performance and long‐term safety of degradable metallic implants requires strict design rules that fit each application. Figure 7 summarizes the main criteria for cardiovascular stents and orthopedic fixation devices across four dimensions: mechanical properties, biocompatibility, corrosion behavior and mechanical integrity during resorption. Both device types demand high mechanical strength (e.g., tensile strength >300 MPa), yet their specific requirements differ because of the distinct physiological environments. Ideally, orthopedic implants should possess an elastic modulus close to that of cortical bone to minimize stress shielding, while concurrently supporting osteoblast adhesion and growth [50]. In contrast, cardiovascular stents focus on suppressing smooth‐muscle proliferation and achieving full resorption within 1–2 years [51].

Unlike cardiovascular stents, which can be metabolically cleared within a relatively short period, orthopedic implants are required to provide sustained and precisely calibrated structural support throughout the bone‐healing process [1]. This distinction is critical, because for orthopedic fixation devices and other structural implants, clinical performance depends not only on the initial load‐bearing capacity, but also on the ability to maintain adequate mechanical support over the entire healing period. Under physiological cyclic loading, Zn‐based implants may be particularly susceptible to stress‐assisted localized degradation. The formation of corrosion pits can act as severe stress concentrators, leading to a non‐linear and potentially catastrophic reduction in fatigue resistance and compressive strength long before overall degradation is complete [37]. In addition, the degradation trajectory is highly dynamic. The transient accumulation of dense corrosion products within the implant structure, such as Zn phosphate, may in some cases induce anomalous short‐term mechanical stabilization, thereby further complicating the prediction of the structural evolution [52, 53]. For load‐bearing applications, implant design should not rely solely on initial mechanical properties and average corrosion rate. It is necessary to establish explicit relationships between degradation time and mechanical property retention, including residual strength, fatigue behavior, fracture mode evolution, and the associated failure risk of Zn‐based implants.

### 3.1 Corrosion Behavior and Degradation Control

**FIGURE 8:**
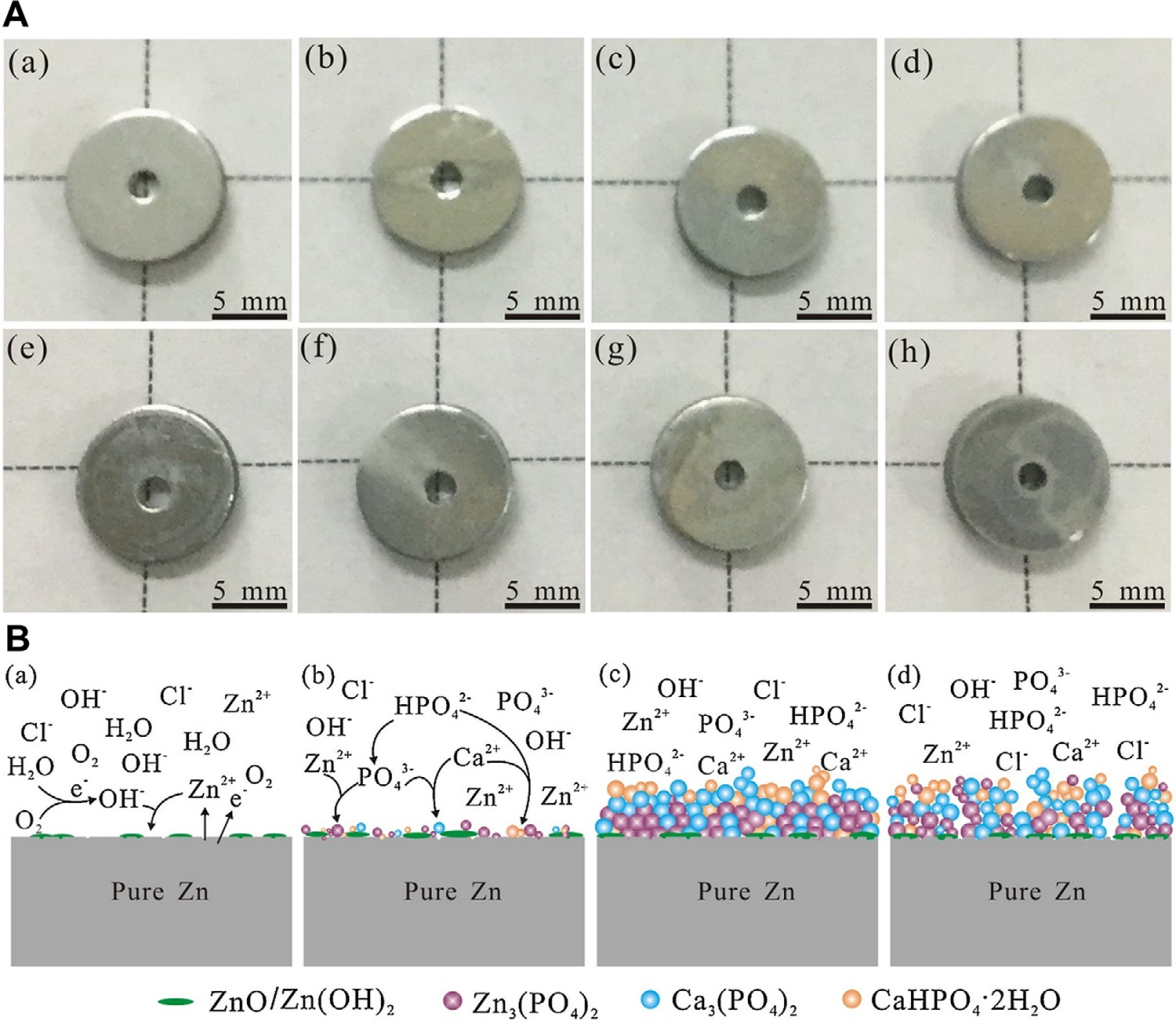
Time‐dependent corrosion evolution of pure Zn in simulated physiological environments. (A) Optical images of pure Zn tested in r‐SBF over time. (B) Schematic of Zn corrosion in r‐SBF [60]. Copyright 2018, Elsevier.

Zn is generally considered to exhibit an intermediate degradation behavior among biodegradable metals, but its corrosion characteristics are strongly influenced by the surrounding medium, pH, and exposure time [54, 55]. Chen et al. [53] examined the corrosion of Zn, Fe, and Mg in phosphate‐buffered saline (PBS) through both electrochemical and immersion tests. They found that, in transient electrochemical measurements, the corrosion rate of Zn was between that of Mg and Fe; however, during long‐term immersion, Zn exhibited localized corrosion, resulting in a faster overall corrosion rate compared to Mg and Fe. Dong et al. [54] evaluated the corrosion behavior of Zn, Mg, and Fe in DMEM and SBF. Zn's degradation rate was significantly slower in DMEM than in SBF. Instead, the slower degradation in DMEM is mainly associated with the presence of abundant organic components including amino acids, glucose and vitamins, which can adsorb onto the Zn surface to form a thin protective layer that restricts interfacial charge transfer and ionic diffusion [56, 57]. Meanwhile, DMEM relies on a physiological NaHCO_3_/CO_2_ buffer system free of Tris‑HCl, whereas Tris‑HCl contained in SBF tends to consume hydroxyl ions generated during corrosion and form complexes with Zn^2+^ and Ca^2+^, thus suppressing the formation of compact and protective surface films [54, 58]. Although Zn forms a Ca‐P deposit layer in SBF, this layer is relatively thin, allowing Cl^−^ to penetrate at defects and cause localized corrosion, thereby affecting the corrosion rate of Zn [59]. Figure 8A [60] shows the corrosion behavior of Zn in r‐SBF at different time intervals. Liu et al. investigated the corrosion behavior of Zn in r‐SBF and pointed out that the corrosion process of pure Zn in r‐SBF (Figure 8B) [60].

The main corrosion mechanism of pure Zn in R‐SBF involves the following reactions [60, 61]

(1) $$\mathrm{Zn}\to Zn^{2+}+2e^{-}$$

(2) $$O_{2}+2H_{2}O+4e^{-}\to 4OH^{-}$$

(3) $$2Zn^{2+}+4OH^{-}\to 2\mathrm{Zn}{\left(\mathrm{OH}\right)}_{2}$$

(4) $$\mathrm{Zn}{\left(\mathrm{OH}\right)}_{2}\to \mathrm{ZnO}+H_{2}O$$

(5) $$\mathrm{HP}{O_{4}}^{2-}+{\mathrm{OH}}^{-}\to {{\mathrm{PO}}_{4}}^{3-}+H_{2}O$$

(6) $$3{\mathrm{Zn}}^{2+}+2{{\mathrm{PO}}_{4}}^{3-}\to {\mathrm{Zn}}_{3}{\left({\mathrm{PO}}_{4}\right)}_{2}$$

(7) $$3{\mathrm{Ca}}^{2+}+2{{\mathrm{PO}}_{4}}^{3-}\to {\mathrm{Ca}}_{3}{\left({\mathrm{PO}}_{4}\right)}_{2}$$

(8) $${\mathrm{Ca}}^{2+}+\mathrm{HP}{O_{4}}^{2-}+2H_{2}O\to \mathrm{CaHP}O_{4}\cdot 2H_{2}O$$

(9) $$\mathrm{Zn}{\left(\mathrm{OH}\right)}_{2}+2Cl^{-}\to Zn^{2+}+2OH^{-}+2Cl^{-}$$

with the cathodic and anodic reactions, Zn^2+^ and OH^−^ are continuously generated and combine to form Zn(OH)_2_. A portion of Zn(OH)_2_ may undergo dehydration to form ZnO. The formation of ZnO can promote the nucleation of phosphate compounds, leading to the formation of Zn_3_(PO_4_)_2_ and Ca_3_(PO_4_)_2_. As the immersion time increases, the phosphates continue to grow, forming abundant calcium phosphate compounds on the top surface of the corrosion layer. As the corrosion products accumulate on the surface of the sample, cracks may appear, leading to a lack of protection for the substrate. This allows Cl^−^ to attack the corrosion products, such as Zn(OH)_2_, further causing the degradation of the metal substrate [32, 60, 62, 63].

As an amphoteric metal, Zn exhibits a characteristic pH‐dependent corrosion behavior, which can be understood through the Pourbaix diagram [64]. In acidic environments, the ZnO/Zn(OH)_2_ passive film is thermodynamically unstable, and Zn dissolves directly as soluble Zn^2+^ ions. The corrosion process is dominated by rapid hydrogen evolution reaction, with sustained anodic dissolution of the zinc matrix and soluble Zn^2+^ as the dominant corrosion product [14, 65].

(10) $$\mathrm{Anodic}\;\mathrm{reaction}:\;\mathrm{Zn}\to Zn^{2+}+2e^{-}$$

(11) $$\mathrm{Cathodic}\;\mathrm{reaction}:\;2H^{+}+2e^{-}\to H_{2}↑$$

(12) $$\mathrm{Zn}{\left(\mathrm{OH}\right)}_{2}+2H^{+}\to {\mathrm{Zn}}^{2+}+2H_{2}O$$

In near‐neutral to mildly alkaline environments, the corrosion products ZnO/Zn(OH)_2_ are thermodynamically stable and can form a relatively protective passive layer on the Zn surface. This material level significantly retards further dissolution, resulting in the lowest corrosion rates within this pH range [59, 66].

(13) $$\mathrm{Anodic}\;\mathrm{reaction}:\;\mathrm{Zn}+2H_{2}O\to \mathrm{Zn}{\left(\mathrm{OH}\right)}_{2}+2H^{+}+2e^{-}$$

(14) $$\mathrm{Cathodic}\;\mathrm{reaction}:\;O_{2}+2H_{2}O+4e^{-}\to 4OH^{-}\cdot$$

In alkaline environments, the ZnO/Zn(OH)_2_ dissolves to form soluble zincate species [Zn(OH)_4_]^2−^, and the metal undergoes accelerated corrosion again [67]:

(15) $$\mathrm{Zn}+4OH^{-}\to {\left[\mathrm{Zn}{\left(\mathrm{OH}\right)}_{4}\right]}^{2-}+2e^{-}$$

(16) $$O_{2}+2H_{2}O+4e^{-}\to 4OH^{-}$$

(17) $$\mathrm{Zn}{\left(\mathrm{OH}\right)}_{2}+2OH^{-}\to {\left[\mathrm{Zn}{\left(\mathrm{OH}\right)}_{4}\right]}^{2-}$$

while the corrosion behavior of Zn is frequently described as “moderate” and compared to Mg, a assessment from a design perspective reveals that its degradation kinetics represent a fundamental paradox, rather than an ideal compromise [3]. Zhao et al. [68]compiled the degradation rates of 130 Zn and Zn‐based alloys and found that the majority of reported values were ≤ 0.05 mm/year. Because the liquid environments in *vivo* and in *vitro* differ substantially, the corresponding degradation rates can also differ and are not directly interchangeable [69]. For example, Ren et al. [70] reported that a Zn‐Cu alloy exhibited a lower degradation rate in *vivo* than in *vitro*. At such low degradation rates, complete resorption may extend well beyond the 12–24 month clinical window targeted for most biodegradable applications [71, 72].

**FIGURE 9:**
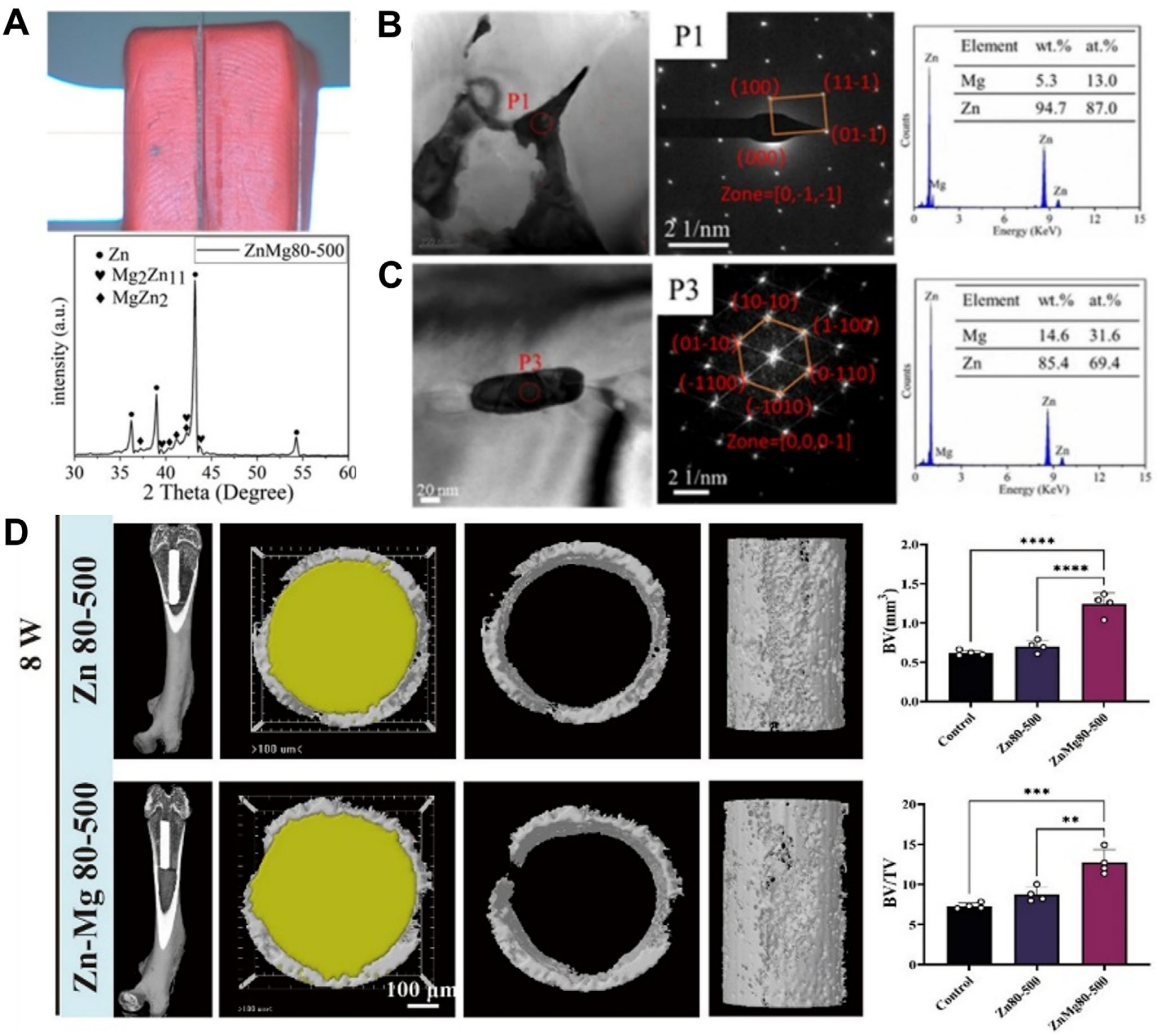
In *vivo* degradation of Zn‐Mg alloys [75]. (A) XRD patterns of Zn‐Mg 80–500 alloy. (B) Mg_2_Zn_11_ alloy phase. (C) MgZn_2_ alloy phase. (D) Micro‐CT quantification at 8 weeks post‐implantation. Copyright 2025, Elsevier.

This “slow degradation dilemma” cannot be resolved by simply accelerating the corrosion rate. Design strategies aimed at increasing degradation, including alloying with highly active elements (e.g., Mg, Li) or increasing surface area via porosity, systematically disrupt Zn's most valuable attribute: its uniform corrosion mode. Such modifications introduce micro‐galvanic couples that promote severe localized pitting corrosion, resulting in a catastrophic and unpredictable loss of mechanical integrity far before complete mass loss is achieved [73, 74]. Consequently, pure Zn degrades too slowly to be clinically relevant, while engineered Zn alloys degrade too heterogeneously to be mechanically reliable. Future design principles must therefore shift from the simplistic goal of “increasing corrosion rate” to the more sophisticated challenge of “achieving controllable, uniform acceleration” without triggering premature mechanical failure. Currently, some studies have achieved controllable degradation rates. Lin et al. [75] used laser additive manufacturing to build a bimodal heterogeneous microstructure in Zn‐Mg alloys (Figure 9A–C). This structure markedly improves mechanical strength and leads to a more uniform and controllable degradation pattern, while also promoting strong bone‐repair performance (Figure 9D).

Most reported corrosion rates for Zn‐based materials have been derived from static immersion tests or potentiodynamic polarization measurements conducted in simulated body fluids under unloaded conditions. Under such protocols, pure Zn typically exhibits relatively uniform corrosion rates of approximately 0.02–0.05 mm/year, which have been widely cited as evidence of an “ideal” and “controllable” degradation profile [3, 22, 60]. However, actual implant environments subject the material to sustained or cyclic mechanical loading, interfacial micromotion, and variable fluid flow. These factors that are not captured by standard static protocols. In Mg‐based biodegradable metals, the synergistic interaction between mechanical stress and corrosive media has been shown to accelerate degradation substantially through stress corrosion cracking and corrosion fatigue [76, 77, 78]. Many Zn alloys exhibit relatively low ductility and fracture toughness compared to Mg alloys [73, 79], which may render them more susceptible to brittle‐type, stress‐assisted crack propagation in corrosive physiological media. Törne et al. [74] further demonstrated that the degradation medium itself significantly alters the corrosion kinetics of Zn, suggesting that even the choice of in *vitro* electrolyte introduces substantial variability. The absence of systematic studies addressing Zn corrosion behavior under physiologically relevant mechanical loading therefore implies that current estimates of implant service life, which are extrapolated from static data, may overestimate the actual structural longevity of Zn‐based devices in load‐bearing applications.

### 3.2 Antimicrobial Properties and Infection Prevention

Implant‐associated infections are a major complication in orthopedic surgery. They can cause surgical failure, require revision procedures and place heavy physical, emotional and economic burdens on patients [80]. Surgical site infections (SSI) account for about 15% of all hospital‐acquired infections, making them the most common type among surgical patients [81]. Reichman [81] reported that *Staphylococcus aureus* and *coagulase‐negative staphylococci* are the major pathogens in fracture surgery, especially in cases involving nails, plates and other fixation devices. Bacterial infections and inflammation that pose serious threats to human health have become a global concern [82]. Therefore, when designing implants, it is crucial to have a thorough understanding of the antibacterial properties of metals/alloys to ensure their optimal performance in application.

**FIGURE 10:**
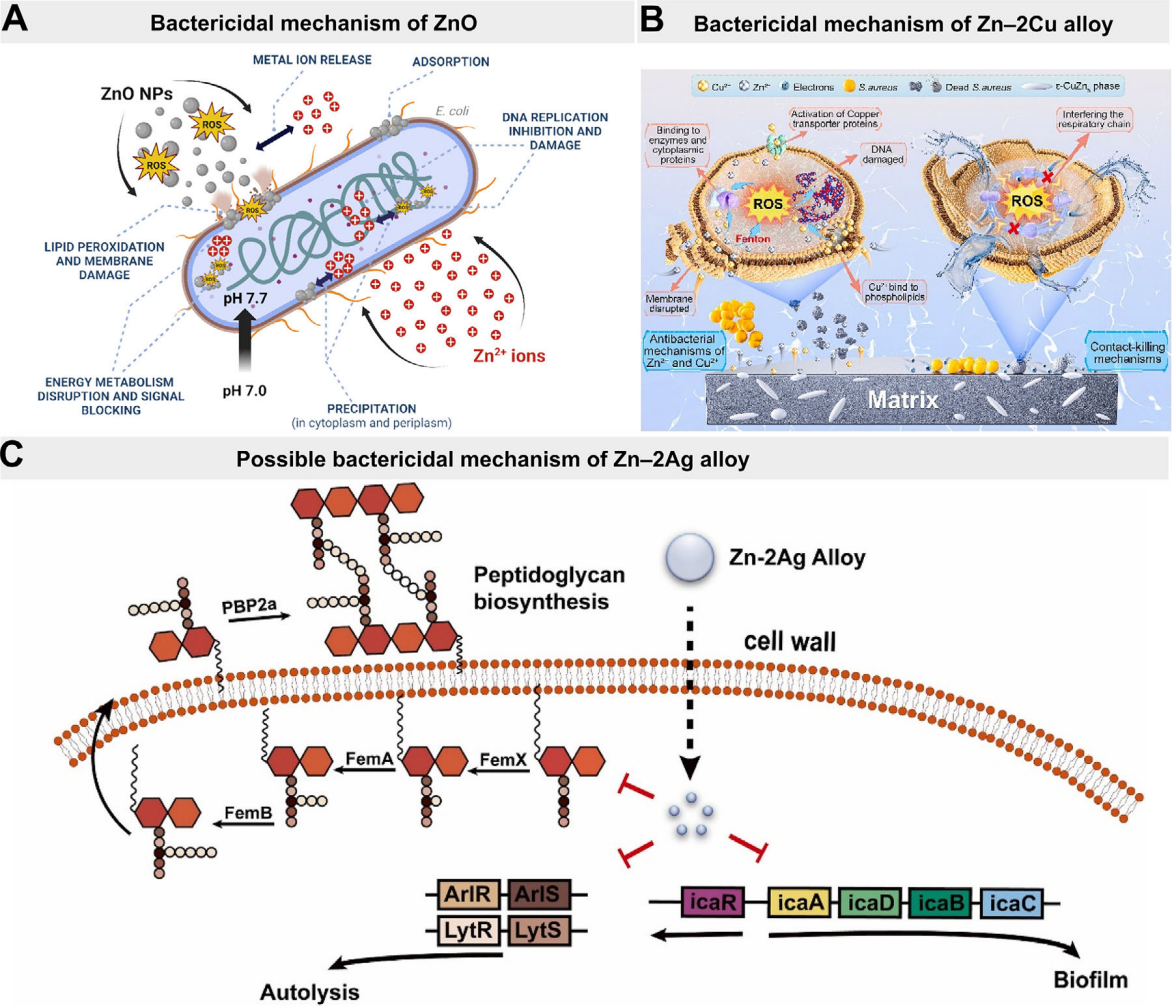
Antibacterial mechanisms of ZnO and Zn alloys. (A) ZnO antibacterial mechanism [83]. Copyright 2024, American Chemical Society. (B) Zn‐2Cu alloy mechanism against MRSA [84]. Copyright 2020, Elsevier. (C) Possible bactericidal mechanism of Zn–2Ag alloy [40]. Copyright 2021, Elsevier.

Zn^2+^ exhibits distinct antibacterial properties. It can directly inhibit bacterial metabolism and block their proliferation, helping to prevent or control infection [85]. Tang et al. [86] found that pure Zn displays weak antibacterial effects because its high corrosion resistance in c‐SBF limits Zn^2+^ release. A key antibacterial mechanism of Zn‐based materials arises from ZnO, which can disrupt bacterial membranes and penetrate into the cytoplasm (Figure 10A) [83]. This process generates high levels of reactive oxygen species (ROS) [83, 87, 88, 89], leading to bacterial death and suppression of biofilm formation. However, pure Zn alone still shows limited ability to prevent infection, adhesion and biofilms. Thus, further enhancement through material modification and related strategies (Figure 10B,C)) [40, 84] is necessary to meet the practical demands of clinical applications [82].

In addition to the intrinsic antibacterial activity associated with Zn^2+^ release, rational structural design may also contribute to antibacterial performance. Biomimetic architectures inspired by natural surfaces have shown potential to inhibit bacterial adhesion and biofilm formation. Zhao et al. [90] developed the Xanthium sibiricum biomimetic Fe‐based medium‐entropy alloy, which achieves simultaneous improvement in antibacterial efficiency and mechanical behaviors precisely through the design of biomimetic nanostructures, confirms the great potential of biomimetic structure design in biomedical antibacterial metallic materials. Although this work did not focus on Zn alloys, it suggests that biomimetic structural design could also be a useful strategy for Zn‐based implants to further enhance antibacterial performance.

### 3.3 Biocompatibility and Tissue Responses

The International Organization for Standardization (ISO) defines biocompatibility as the ability of a material to interact with a biological system without harmful effects, while performing its intended function [14]. It requires that biomaterials be either low in toxicity or non‐toxic, while also being able to elicit appropriate biological responses under specific conditions. This includes tissue and cell compatibility, blood compatibility, immune system compatibility, and mechanical adaptability [91]. Related studies have shown that both Mg alloys and Fe alloys possess good biocompatibility. However, Mg alloys generate hydrogen gas during degradation, leading to gas embolism, while Fe alloys experience severe corrosion, resulting in inflammation issues. As a result, there is a need to explore alternative metals, such as Zn.

**FIGURE 11:**
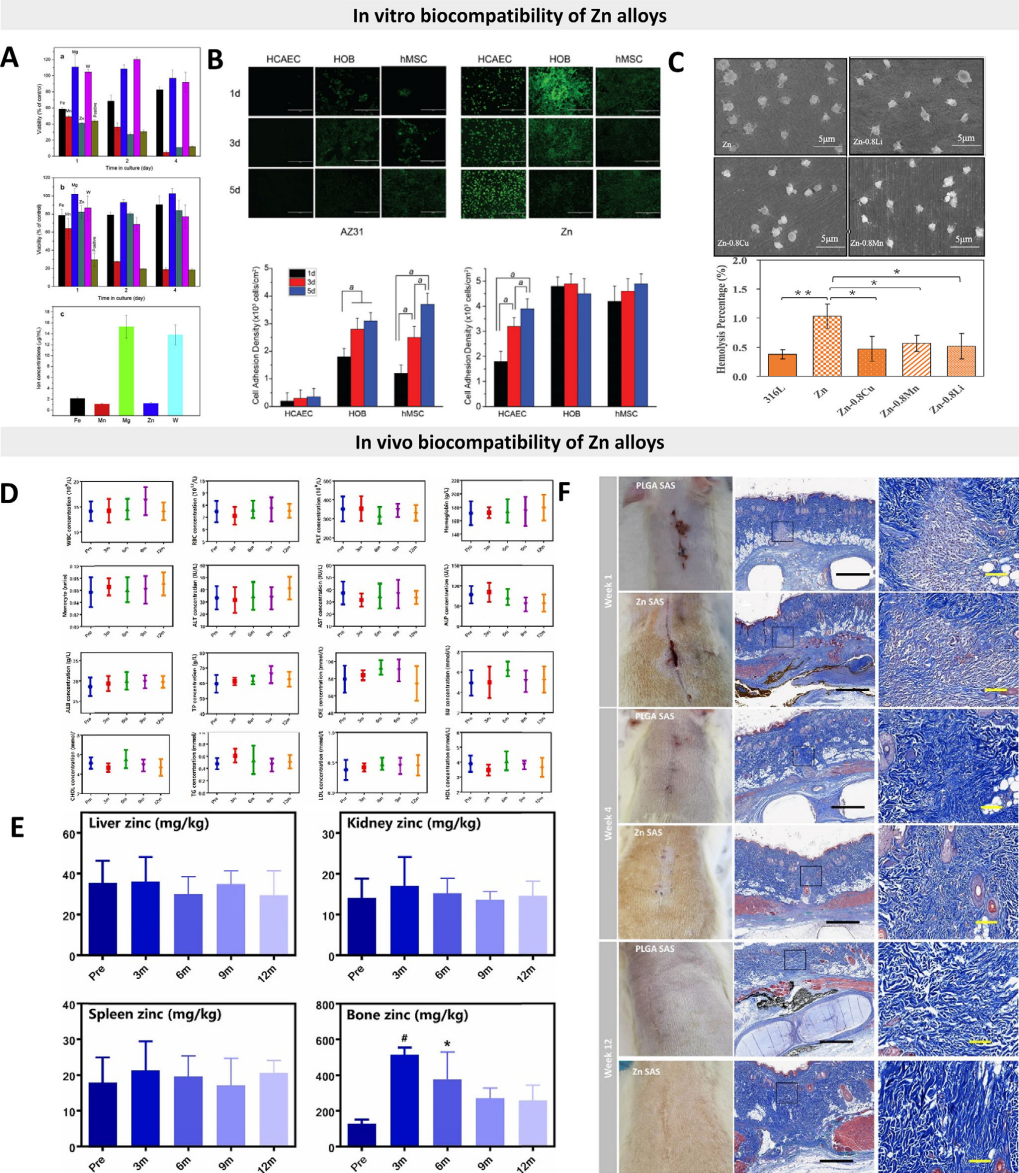
Biocompatibility and tissue responses of Zn‐based implants. (A) Cell viability of L929 and ECV304 cells after 1, 2, and 4 days in extraction media from pure metals (Fe, Mn, Mg, Zn, W) [93]. Copyright 2013, Elsevier. (B) Adhesion of primary HCAECs, HOBs, and hMSCs on AZ31 and Zn surfaces over 1–5 days, with quantitative assessment of cell density [94]. Copyright 2019, American Chemical Society. (C) Cell viability after incubation in 10%, 50%, 75% and 100% Zn alloy extracts for 3 days and corresponding hemolysis ratios [17]. Copyright 2019 Elsevier. (D) Routine blood and biochemical parameters measured before and after Zn‐Mg‐Fe alloy implantation [55]. Copyright 2022 Elsevier. (E) Zn content in liver, kidney, spleen, and adjacent bone following implantation [55]. Copyright 2022 Elsevier. (F) Gross morphology and Masson‐stained histology of wounds closed with PLGA SAS and Zn SAS at 1, 4, and 12 weeks post‐surgery [97]. Copyright 2023 Elsevier.

Cytotoxicity, cytocompatibility, and hemocompatibility tests verify implant biocompatibility. Zinc‐based materials exhibit low toxicity due to physiological Zn^2+^ homeostasis [92]. In 2013, Cheng et al. [93] reported that Zn shows no cytotoxicity to ECV304 cells, though it reduced mouse fibroblast viability (Figure 11A) [93]. Zhu et al. [94] showed superior cytocompatibility of Zn over AZ31 alloy for primary HCAECs, HOBs, and hMSCs (Figure 11B). This superior cytocompatibility can be attributed to several interrelated factors. Previous in vivo studies have demonstrated that the corrosion rate of pure Zn is approximately 15–50 µm/year, an order of magnitude lower than that of pure Mg (300‐600 µm/year) [42, 95]. This slow degradation ensures that the local Zn^2+^ concentration remains within a tolerable and potentially beneficial range, rather than reaching cytotoxic levels [96]. In addition, unlike Mg and its alloys, Zn corrosion does not produce hydrogen gas, thereby avoiding the formation of gas cavities that can disrupt cell adhesion and local tissue architecture [71]. The slow and steady degradation behavior of Zn‐based implants has also been confirmed in long‐term in vivo studies: Zhou et al. [19] showed that Zn‐Cu stents implanted in porcine coronary arteries retained approximately 28 vol% of the original structure even after 24 months, with no signs of inflammation or thrombosis. In terms of hemocompatibility, zinc‐based alloys not only reduce hemolysis rates but also inhibit thrombus formation. Yin et al. [17] investigated the hemocompatibility of binary Zn‐0.8 wt.% Cu, Mn and Li alloys. All three alloys showed a hemolysis rate below 1%, and exhibited distinctly lower platelet adhesion than pure Zn (Figure 11C).

In addition to the aforementioned evaluations of the in *vitro* biocompatibility of Zn, researchers have also conducted extensive investigations into its in *vivo* biocompatibility. They fabricated pure Zn or Zn alloys into wires, screws, or stents and implanted them into animals (mice, rabbits, or dogs) to assess their biocompatibility in *vivo*. Shao et al. [55] systematically investigated the in *vivo* biocompatibility of a Zn‐Mg‐Fe alloy in beagle dogs. Hematological and blood biochemical analyses were performed before and after implantation, and indices such as WBC, RBC, and ALT were found to remain within normal ranges (Figure 11D) [55]. After implantation, the Zn^2+^ content in organs such as the liver, kidneys, and spleen was monitored, and no accumulation of Zn^2+^ was detected in these organs (Figure 11E) [55], indicating that it was metabolized. Zn‐1.0Cu‐0.5Ca subcuticular staples also demonstrated good biocompatibility in rats [97]. At 12 weeks post‐operation, the wound was nearly fully healed, with a healing capacity comparable to that of PLGA (PLA‐PGA copolymer) (Figure 11F) [97], and no obvious inflammation was observed. Therefore, Zn exhibited good biocompatibility in both in *vivo* and in *vitro* experiments, indicating its suitability for use as an implant material.

Despite the generally favorable biocompatibility reported for Zn, considerable inconsistencies persist in the literature regarding the cytotoxic threshold of Zn^2+^ ions. For example, Murni et al. [98] reported that Zn‐3Mg alloy extracts at relatively high concentrations remained compatible with normal human osteoblast cells, and Kubásek et al. [99] similarly found acceptable cytocompatibility of Zn‐Mg alloys toward U‐2 OS osteosarcoma cells. In contrast, Shearier et al. demonstrated that human aortic endothelial cells and smooth muscle cells exhibited significantly impaired adhesion and proliferation when exposed to Zn^2+^ levels as low as 50 µM [100], and Ma et al. [101] confirmed dose‐dependent cytotoxicity of Zn toward endothelial cells. These contradictory thresholds arise in part from intrinsic differences in cell‐type sensitivity: bone‐related cells appear substantially more tolerant to Zn^2+^ than vascular cells, which has implications for site‐specific implant design [7]. Additionally, the choice of degradation medium has been shown to alter both the rate and speciation of Zn^2+^ release in *vitro* [102], while Yamamoto and Hiromoto [103] demonstrated that proteins and amino acids substantially modify degradation behavior of metallic biomaterials. Extrapolating these in *vitro* safe thresholds to in *vivo* scenarios is therefore questionable, because the local concentration of Zn^2+^ in the immediate vicinity of an implant may transiently exceed static in *vitro* limits [16, 79].

### 3.4 Biological Functionality

#### 3.4.1 Immunomodulatory Effects

**FIGURE 12:**
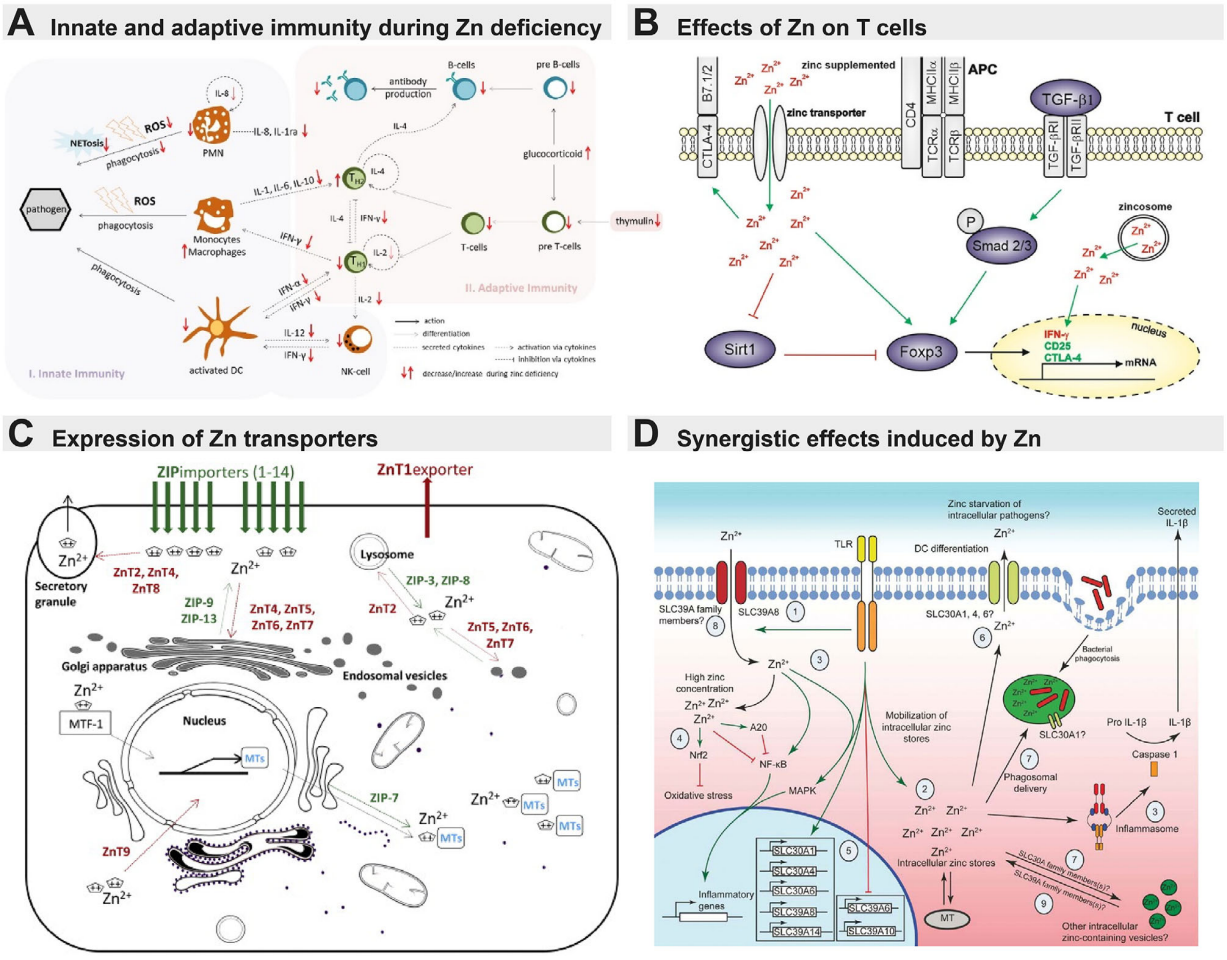
Zn‐dependent immune regulation. (A) Overview of innate and adaptive immune cells and their interactions under zinc deficiency [105]. Copyright 2016, Elsevier. (B) Molecular mechanisms regulating Treg cell function [108]. Copyright 2017, Medicine. (C) Subcellular localization of human ZIP/ZnT transporters and Zn homeostasis regulator [104]. Copyright 2015, Elsevier. (D) Relationship between Zn trafficking, inflammatory signaling, and antimicrobial responses in macrophages [110]. Copyright 2013, Portland Press Ltd.

Zn is a vital trace element essential for immune function. It modulates both innate and adaptive immunity by regulating cellular homeostasis, signaling pathways, and cytokine production (Figure 12A) [104, 105]. In innate immunity, Zn supports neutrophil chemotaxis, phagocytosis, and formation of neutrophil extracellular traps (NETs), all critical for pathogen clearance [106]. It also promotes monocyte differentiation into macrophages and regulates their phagocytic activity and secretion of pro‐inflammatory cytokines, including TNF‐α and IL‐1β [104, 107]. In addition, Zn improves the cytotoxic activity and differentiation of natural killer (NK) cells by tuning the interaction between inhibitory receptors and MHC I, thus reducing unintended cell killing [28, 106].

In adaptive immunity, Zn is required for both the development and function of T cells. Zn deficiency causes thymic atrophy, increases apoptosis of pre‐T cells and results in reduced T‐cell numbers (Figure 12B) [108]. It also disrupts the Th1/Th2 balance by sharply lowering the secretion of Th1 cytokines such as IFN‐γ and IL‐2, while having a milder effect on Th2 function [105, 109].

The immunoregulatory effects of Zn depend on precise molecular mechanisms and tight homeostatic control. As shown in Figure 12C [104], intracellular Zn levels are maintained by ZIP transporters (e.g., ZIP6, ZIP8) and ZnT transporters (e.g., ZnT5, ZnT10). Metallothioneins act as intracellular Zn buffers by binding and releasing Zn as needed [104, 106]. At the signaling level, Zn can inhibit enzymes such as protein tyrosine phosphatases (PTPs) and phosphodiesterases (PDEs), thereby adjusting cAMP/cGMP concentrations and controlling NF‐κB activation [106, 107]. For example, Zn suppresses NF‐κB signaling by inducing A20 expression, which in turn lowers the production of pro‐inflammatory cytokines (Figure 12D) [110].

Related experimental studies have shown that Zn‐based alloy implants can suppress local inflammation and avoid severe immune responses, demonstrating high safety. Compared to pure Ti, rats implanted with Zn‐2Cu alloy exhibited only mild infiltration of inflammatory cells in the bone marrow cavity, with no abscess formation, and normal serum inflammation markers [84]. Similarly, rats implanted with Zn‐Al alloy in the aorta for 1 month showed only mild fibrosis in the adventitia, with no acute inflammation or necrosis [3].

#### 3.4.2 Osteogenic and Angiogenic Effects

**FIGURE 13:**
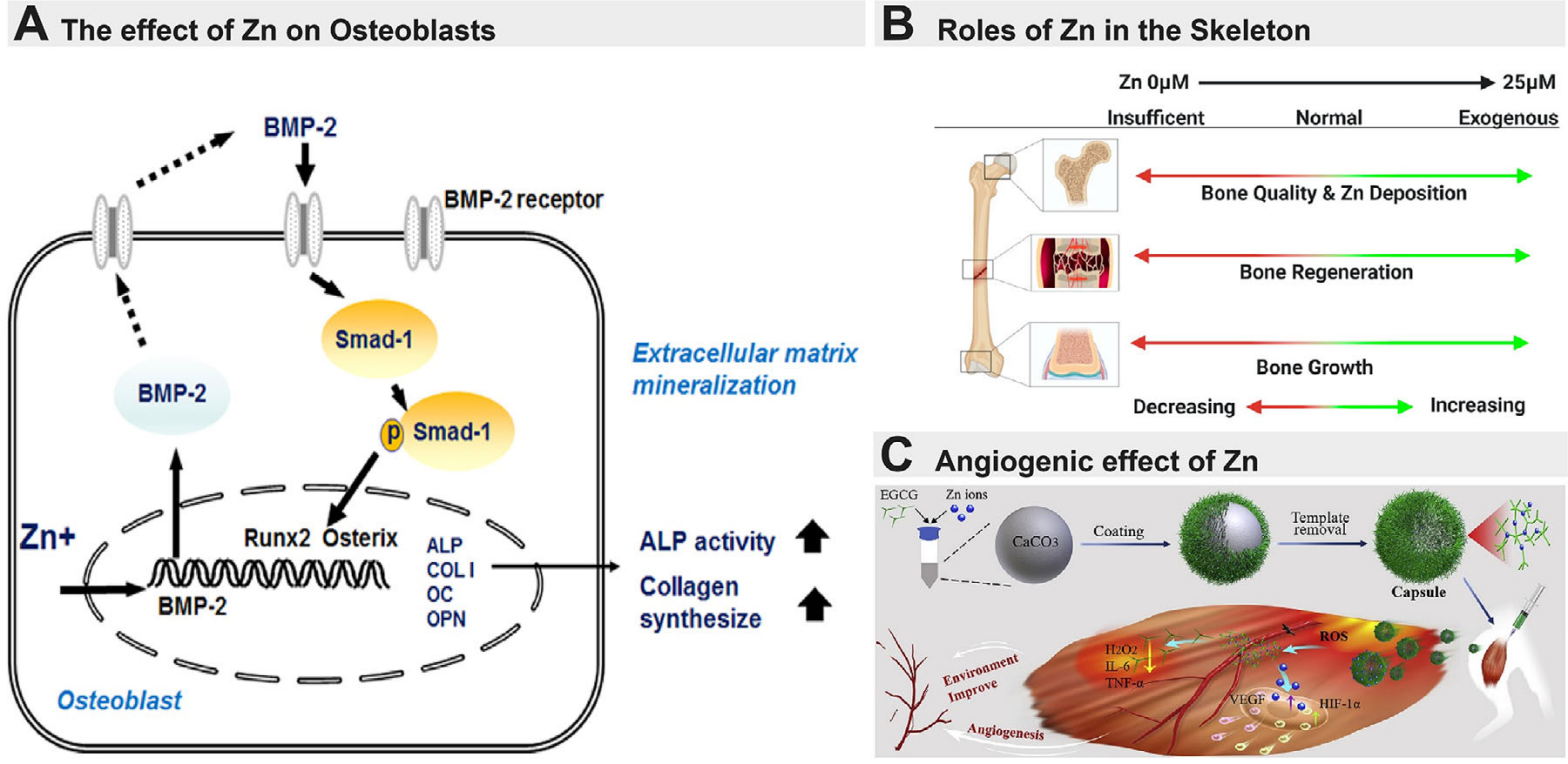
Zn‐mediated osteogenic and angiogenic effects. (A) Proposed mechanism of zinc regulation via BMP‐2 signaling in osteoblasts [113]. Copyright 2018, The Korean Nutrition Society. (B) Roles of Zinc in the Skeleton [112]. Copyright 2020, MDPI. (C) ROS‐responsive EGCG‐Zn capsules enhancing therapeutic angiogenesis in a mouse model of limb ischemia [116]. Copyright 2021, Elsevier.

Zn is an essential component of the bone matrix [111]. As shown in Figure 13A,B [112, 113], Zn‐based implants can exhibit significant osteogenic activity both in vitro and in vivo by upregulating osteogenic genes and enhancing mineralization capacity [114]. One month after implanting Zn alloy into the femoral condyle of rats, Van Gieson staining revealed significant new bone formation around the implant. The bone cells were evenly distributed, and there was no apparent fibrous gap between the new bone and the implant [94]. Ji et al. [115] investigated a Zn‐2Cu‐0.5Zr alloy and demonstrated that Zn^2+^ markedly enhances bone repair in age‐related osteoporotic fractures. This effect is mediated through modulation of macrophage polarization, increased secretion of the anti‐inflammatory cytokine IL‐10, and suppression of pro‐inflammatory cytokines TNF‐α and IL‐6.

Zn^2+^ is an activator of vascular endothelial growth factor, promoting angiogenesis (Figure 13C) [116] and inhibiting thrombosis, without causing significant vascular damage [3, 117]. The hemolysis rate of Zn‐Cu‐Fe alloy is approximately 1% (well below the 5% safety threshold). Platelets maintained an oval shape on the material surface, with no aggregation or deformation observed, indicating excellent antithrombotic properties.

## 4 Materials and Surface Engineering Strategies

### 4.1 Alloy Design

Pure Zn exhibits excellent biocompatibility, but its mechanical properties are relatively poor, which limits its suitability for most biomedical applications. An effective solution is to introduce functional metals such as Mg, Mn, Fe, Li, Cu, and Ag through alloying, thereby achieving synergistic enhancement of their properties [37, 82]. The following summarizes some of the research findings by other scholars (Table 1).

**TABLE 1:** Mechanical properties and corrosion rate of Zn alloys.

Alloy	UTS (MPa)	YS (MPa)	E (%)	CR (mm/year)	Biocompatibility	References
Pure Zn	100	50	8	0.6 (Electrochemical) 0.05 (Modified Hanks’ solution soaked for 3 months)	Endothelial cell viability: 90% bone contact rate (BIC): 20%	[118]
Zn‐0.4Fe	150	100	30	Slightly lower than pure Zn	Endothelial cell viability: 98% BIC: 65%	
Zn‐2.5Fe	150	100	10	Slightly lower than pure Zn	Endothelial cell viability: 100% BIC: 10%	
As‐cast pure Zn	33.6±0.68	29.3 ± 0.3	1.2 ± 0.3	0.1567 ± 0.0117 (Electrochemical) 0.068 (Hank's solution soaked for 14 days)	Day 3: The cell viability of MC3T3‐E1 in an extraction solution with 25% concentration was approximately 65.43±1.57%	[119]
As‐cast Zn‐5Ge	53.9 ± 0.7	47.5 ± 1.7	1.1 ± 0.2	0.1272 ± 0.0132 (Electrochemical) 0.042 (Hank's solution soaked for 14 days)	Day 3: The cell viability of MC3T3‐E1 in an extraction solution with 25% concentration was approximately 77.66 ± 0.59%	
Hot rolling pure Zn	153.1 ± 3.2	84.2 ± 2.9	61.4 ± 1.2	0.3057 ± 0.0190 (Electrochemical) 0.099 (Hank's solution soaked for 14 days)	—	
Hot rolling Zn‐5Ge	237.0 ± 3.4	175.1 ± 1.8	21.6 ± 2.8	0.2255 ± 0.0146 (Electrochemical) 0.051 (Hank's solution soaked for 14 days)	—	
Zn‐1% Mg	—	—	—	0.012 ± 0.002 (Electrochemical)	The viability of Saos‐2 cells exceeded 100% after 24‐h and 48‐h culture with extract solutions.	[120]
Zn‐1% Mg‐0.5% Ca	—	—	—	0.066 ± 0.004 (Electrochemical)	The viability of Saos‐2 cells exceeded 100% after 24‐h and 48‐h culture with extract solutions.	
Zn‐2Li	360	—	18	0.06 (Electrochemical)	—	[121]
Zn‐4Li	420	—	15	0.05 (Electrochemical)	—	
Indirectly extruded Pure Zn	65	35	3.7	0.653 (Electrochemical)	No toxicity was observed in murine fibroblasts (L‐929). In *vivo* (rabbit model, 4–24 weeks): No inflammatory cells were detected, and new bone formation was observed at the implant interface.	[122]
Indirectly extruded Zn‐0.05Mg	225	160	26	0.728 (Electrochemical)	Non‐toxic to L‐929 and exhibits potent antibacterial activity. In *vivo* (rabbit model, 4–24 weeks): No inflammatory response observed within 24 weeks, promoting new bone formation at the bone/implant interface.	
Zn‐3Fe‐1Si	156.5	99.6	40	0.012 ± 0.006 (0.9% NaCl solution soaked for 2 weeks)	HUVEC cells: Survival rate of approximately 80% on day 1; complete proliferation to approximately 110% by day 3.	[123]
Zn‐6Fe‐6Si	133.6	86.7	30	0.016 ± 0.009 (0.9% NaCl solution soaked for 2 weeks)	HUVEC cells: Survival rate was approximately 50% on day 1, and activity fully recovered to about 100% by day 3.	
Zn‐5Cu	160.1	—	—	0.653 (Electrochemical)	No significant cytotoxicity was observed in BMSCs (OD value of approximately 0.28 on day 1 with 10‐fold diluted extract, increasing to about 0.61 on day 3).	[124]
Zn‐5Cu‐0.5Mg	175.5	—	—	0.703 (Electrochemical)	BMSCs exhibited favorable cell proliferation (OD value approximately 0.28 on day 1, increasing to about 0.60 on day 3)	
Zn‐5Cu‐1Mg	187.3	—	—	0.718 (Electrochemical)	BMSCs exhibited favorable cell proliferation status (OD value approximately 0.28 on day 1, increasing to about 0.62 on day 3)	
Zn‐5Cu‐2Mg	216	—	—	0.728 (Electrochemical)	BMSCs exhibited favorable cell proliferation status (OD value approximately 0.29 on day 1, increasing to about 0.67 on day 3). In *vivo* (subcutaneous injection at day 7): M2 anti‐inflammatory macrophages demonstrated the highest expression levels.	
Extruded Pure Zn	102.02 ± 1.39	67.39 ± 1.28	—	0.479 (Electrochemical)	In *vitro*: MC3T3‐E1 showed stable proliferation (increased to approximately 2.5 by day 7). In *vivo* (rat mandible, 4–8 weeks): At 4 weeks, the IOD/area ratio of newly formed bone was approximately 0.06.	[125]
Extruded Zn–2Cu	385.56 ± 2.47	298.45 ± 1.89	—	0.612 (Electrochemical)	In *vitro*: Normal proliferation of osteoblasts (OD value approximately 2.6 on day 7) In *vivo*: New bone IOD/area at 8 weeks approximately 0.065	
Extruded Zn–2Cu–0.8Li	517.33 ± 3.66	422.82 ± 1.69	—	0.463 (Electrochemical)	In *vitro*: Osteoblast proliferation was favorable, with the strongest mineralization‐promoting capacity. In *vivo*: New bone reached its peak at 8 weeks (IOD/area approximately 0.09).	
Zn	111 ± 4.5	51 ± 3.7	60 ± 5.9	0.25 (Electrochemical)	—	[126]
Zn–0.15Mg	250 ± 9.2	114 ± 7.7	22 ± 4.0	0.30 (Electrochemical)	—	
Zn–0.5Mg	297 ± 6.5	159 ± 8.5	13 ± 0.9	0.31 (Electrochemical)	—	
Zn–1Mg	340 ± 15.6	180 ± 7.3	6 ± 1.1	0.32 (Electrochemical)	—	
Zn–3Mg	399 ± 14.4	291 ± 9.3	1 ± 0.1	0.24 (Electrochemical)	—	
Zn–0.5Al	203 ± 9.6	119 ± 2.3	33 ± 1.2	0.28 (Electrochemical)	—	
Zn–1Al	223 ± 4.3	134 ± 5.8	24 ± 4.2	0.29 (Electrochemical)	—	
Zn	98.5 ± 3.2	48.8 ± 2.1	9.8 ± 1.1	0.196 ± 0.015(Hanks’ solution soaked for 3 months)	The activity of Human Umbilical Vein Endothelial Cell Line EA.hy926 is approximately 98%.	[127]
Zn‐0.5Cr	198.6 ± 4.5	118.3 ± 3.5	19.6 ± 1.8	0.278 ± 0.020(Hanks’ solution soaked for 3 months)	The cell activity was maintained at ∼95%, with a bactericidal rate of approximately 59%.(*E. coli*)	
Zn‐0.5Zr	201.2 ± 5.1	138.7 ± 4.2	24.8 ± 2.0	0.147 ± 0.012((Hanks’ solution soaked for 3 months)	The cell viability was approximately 94%, and the bactericidal rate was about 58%.(*E. coli*)	
Zn‐0.5V	203.5 ± 5.5	158.9 ± 4.8	29.5 ± 2.3	0.126 ± 0.010((Hanks’ solution soaked for 3 months)	The cell viability was approximately 10%, with a bactericidal rate of about 65%.(*E. coli*)	
ZM (Zn‐0.1Al‐0.1Mn‐0.1Cu‐0.1Ag)	207 ± 0.9	154 ± 1.7	82.2 ± 2.94	0.241 ± 0.004 (Electrochemical)	—	[128]
ZM‐0.1Li	380 ± 4.6	329 ± 1.6	49.4 ± 2.64	0.206 ± 0.006 (Electrochemical)	—	
ZM‐0.35Li	449 ± 7.4	380 ± 1.6	62.3 ± 4.63	0.189 ± 0.008 (Electrochemical)	—	
Pure Zn	160 ± 16	120 ± 12	40 ± 4	0.015 (Electrochemical)	The antibacterial efficacy was weak, with surface MRSA/MRSE colony counts remaining as high as approximately 1000–1400 CFUs. In *vivo*: In a murine cranial osteolysis model, the bone volume/total volume ratio (BV/TV) was approximately 45%, and bone mineral density (BMD) was about 90 mg/cc.	[40]
Zn‐0.5Ag	170 ± 17	125 ± 12	38 ± 4	0.026 (Electrochemical)	The surface antibacterial activity was moderate, with a certain reduction in colony count compared to pure zinc.	
Zn‐1Ag	190 ± 19	135 ± 13	65 ± 7	0.019 (Electrochemical)	After 24 h of co‐culture, the surface MRSA/MRSE colony counts significantly decreased to levels approaching zero.	
Zn‐2Ag	230 ± 23	180 ± 18	35 ± 4	0.016 (Electrochemical)	The surface exhibits potent bactericidal activity, with a pathogenic colony count of 0 in *vivo* (mice at 14 days): BV/TV: approximately 60%, bone porosity 35%.	

Abbreviations: CR: Corrosion Rate; E: Elongation; UTS: Ultimate Tensile Strength; YS: Yield Strength.

**FIGURE 14:**
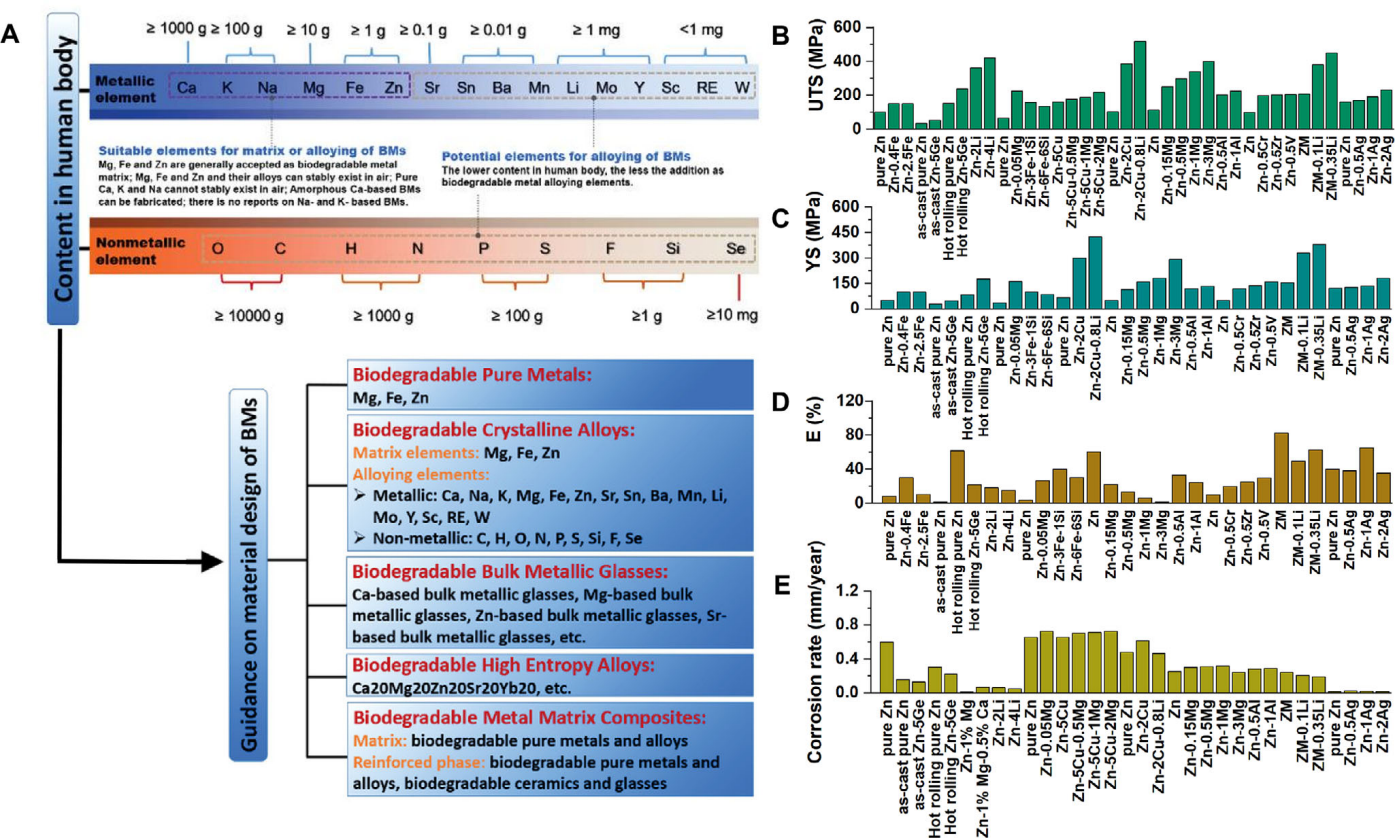
Alloying strategies for tuning Zn properties. (A) Design of biodegradable metals through elemental selection [14]. Copyright 2019, John Wiley and Sons. (B) The Ultimate tensile strength, (C) yield strength, (D) elongation, and (E) electrochemical corrosion rate of Zn alloys in relevant literature [40, 118, 119, 120, 121, 122, 123, 124, 125, 126, 127, 128].

From the perspective of elemental abundance in the human body, Ca, K, and Na are the most prevalent. However, according to the periodic table, Na and K belong to the first main group (Group IA) and possess high chemical reactivity. Consequently, their application in implant materials has not yet been established. Therefore, a multi‐dimensional assessment is required when selecting additional elements for biodegradable metal implants. Relevant researchers have summarized candidate elements from the periodic table suitable for these applications and, aiming at future research directions, have proposed five distinct groups of biodegradable metals (Figure 14A) [14]. Reported mechanical properties and corrosion rates of various zinc‐based alloys are summarized in Figure 14B–E.

#### 4.1.1 Zn‐Mg Alloy

**FIGURE 15:**
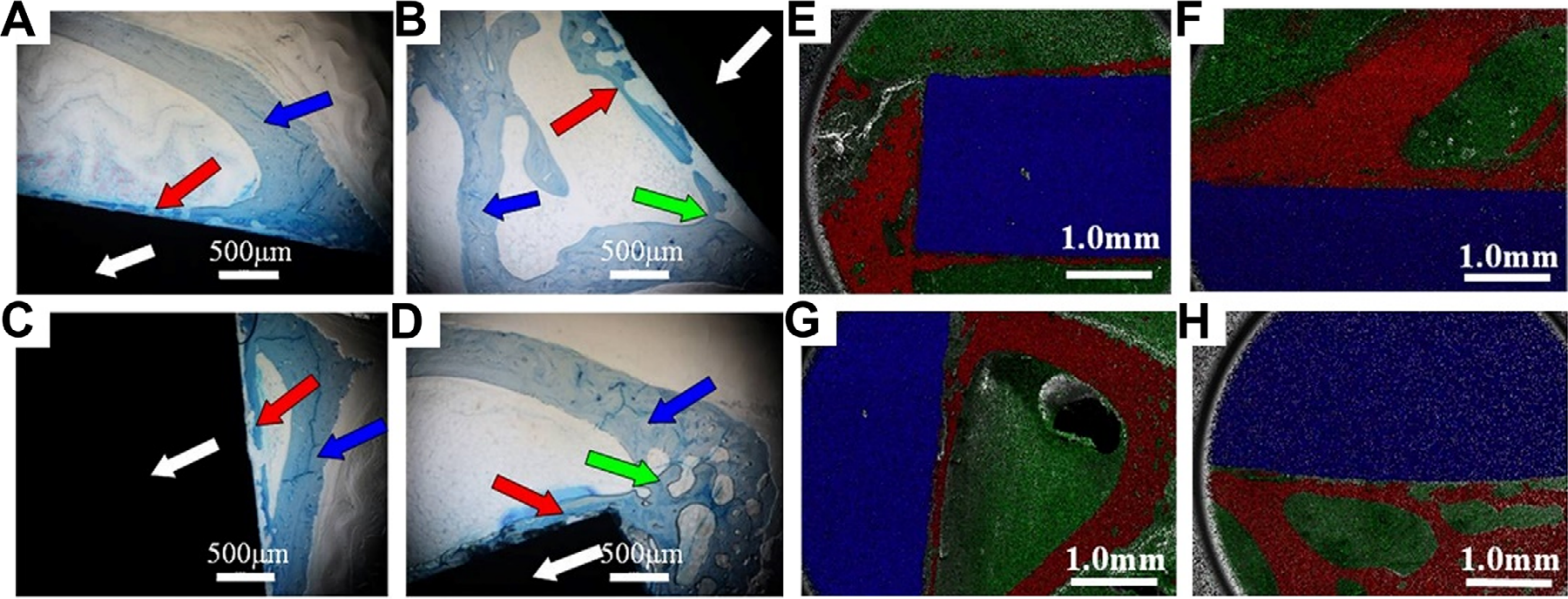
Microstructure and performance of Zn–Mg alloys [122]. Representative histology of bone‐implant cross sections stained with Toluidine blue: Zn for (A) 12 weeks, (B) 24 weeks; Zn‐0.05Mg for (C) 12 weeks, (D) 24 weeks. Elemental mapping of the bone‐implant interface: Zn at (E) 12 weeks and (F) 24 weeks; Zn‐0.05Mg for (G) 12 weeks and (H) 24 weeks. Copyright 2018, Elsevier.

Mg, an essential light metal element for the human body with a recommended daily intake of 350 mg, can be alloyed with Zn to modulate both the mechanical properties and osteogenic potential of the alloy [129, 130]. When the Mg content is low, Mg atoms can dissolve into the Zn lattice to form a solid solution, Solid solution strengthening originates from the interplay of solute atoms and dislocations; solute atoms induce lattice distortion, which impedes dislocation movement and thereby produces a strengthening effect, increasing tensile strength through solid‐solution strengthening [131, 132]. When additional Mg is present, it reacts with Zn to form intermetallic phases such as MgZn_2_ and Mg_2_Zn_11_. These nano‐precipitates effectively pin grain growth during dynamic recrystallization (DRX), and serve as strengthening particles distributed along grain boundaries and within the matrix, further raising the alloy's strength and hardness through second‐phase strengthening [133]. While Mg substantially improves tensile strength, it reduces elongation; for instance, Zn‐3Mg exhibits a UTS of 399 MPa and a YS of 291 MPa, but elongation is limited to 1% [126]. The Mg_2_Zn_11_ phase also serves as a corrosion barrier, enhancing the corrosion resistance of Zn‐5Al‐2Mg alloys [134]. In porous scaffolds with 1% Mg, weight loss reached 7.01% after 28 days of immersion. Due to Mg's higher reactivity compared to Zn, Mg^2+^ concentration in the alloy exceeds that of Zn^2+^. Mg^2+^ stimulates local neuronal release of calcitonin gene‐related peptide‐α (CGRP), promoting bone fracture healing [135]. Xiao et al. [122] implanted Zn and Zn‐0.05 wt.% Mg alloys into the bilateral tibial shafts of New Zealand rabbits. Histological analysis of the implantation site (Figure 15A–D) revealed new bone formation within 12 weeks, with close contact and fusion between the implant and newly formed bone. High‐density Ca was observed around the cortical bone and implant, indicating effective osteogenesis induced by both Zn and Zn‐0.05Mg alloys (Figure 15E–H).

#### 4.1.2 Zn‐Mn Alloy

**FIGURE 16:**
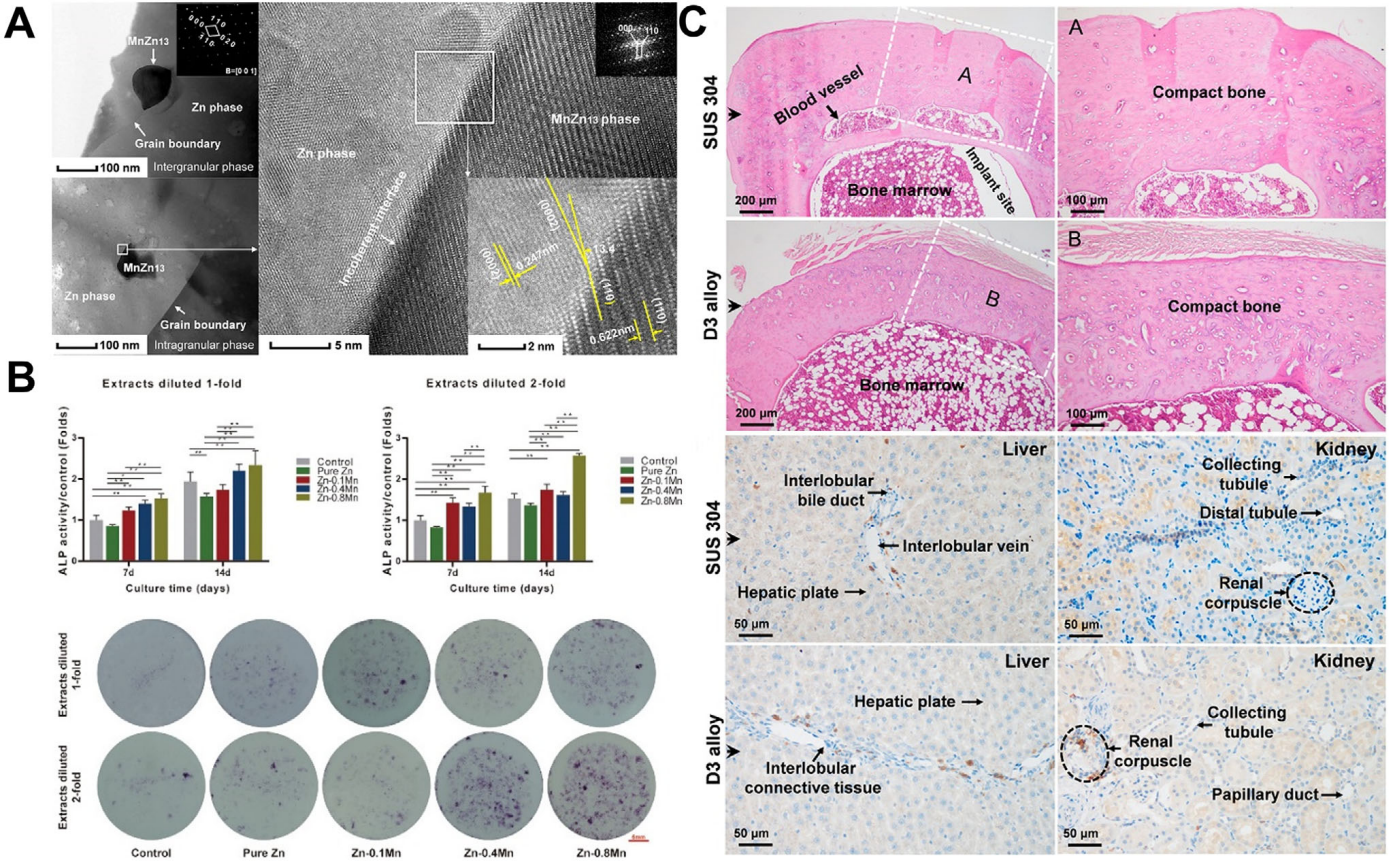
Microstructure and performance of Zn–Mn alloys. (A) TEM, SAED and HRTEM images of the interface between Zn phase and MnZn_13_ phase in Zn‐0.5Mn alloy [139]. Copyright 2021, Elsevier. (B) ALP activity and staining of MC3T3‐E1 cells cultured in osteogenic medium with pure Zn and Zn‐Mn alloy extracts [140]. Copyright 2020, Elsevier. (C) Optical images of H&E‐stained bone sections and CD31 immunohistochemistry after 120 days of Zn‐0.5Mn implantation [139]. Copyright 2021, Elsevier.

Mn is an essential trace element involved in glucose and lipid metabolism as well as cartilage synthesis [136]. Adding small amounts of Mn to Zn effectively refines grain structure and enhances alloy strength [137]. Both cast and hot‐rolled Zn‐Mn alloys contain a secondary MnZn_13_ phase, whose quantity increases with Mn content [138]. From a metallurgical perspective, these MnZn_13_ intermetallic particles act as effective obstacles that exert a Zener pinning effect on grain boundaries, thereby stabilizing small‐angle boundaries and significantly inhibiting grain growth during thermal processing (Figure 16A) [137, 139]. This grain refinement contributes to the overall mechanical integrity of the alloy by increasing the yield strength in accordance with the Hall‐Petch relationship [139]. Furthermore, the fine and dispersed MnZn_13_ precipitates function as hard sites that hinder dislocation movement, providing a significant dispersion‐strengthening component that compensates for the inherent softness of pure Zn [137]. Bo et al. [140] implanted Zn‐0.8Mn alloys and pure Ti scaffolds into rat femoral condyles to repair bone defects. Prior to implantation, ALP activity induced by alloy extracts on MC3T3‐E1 osteogenic differentiation was quantified (Figure 16B). Post‐surgery histology showed abundant new bone formation around Zn‐0.8Mn implants, demonstrating strong bone repair. Mn plays a key role in bone metabolism by upregulating osteogenic genes such as ALP and BMP, promoting collagen regeneration, regulating bone remodeling, and maintaining bone mass [141, 142]. Guo et al. [139] implanted Zn‐0.5Mn alloy into rat tibias. Histological analysis indicated that the implant supported marrow recovery and enhanced hematopoietic function, confirming Mn's osteogenic contribution (Figure 16C).

#### 4.1.3 Zn‐Fe Alloy

**FIGURE 17:**
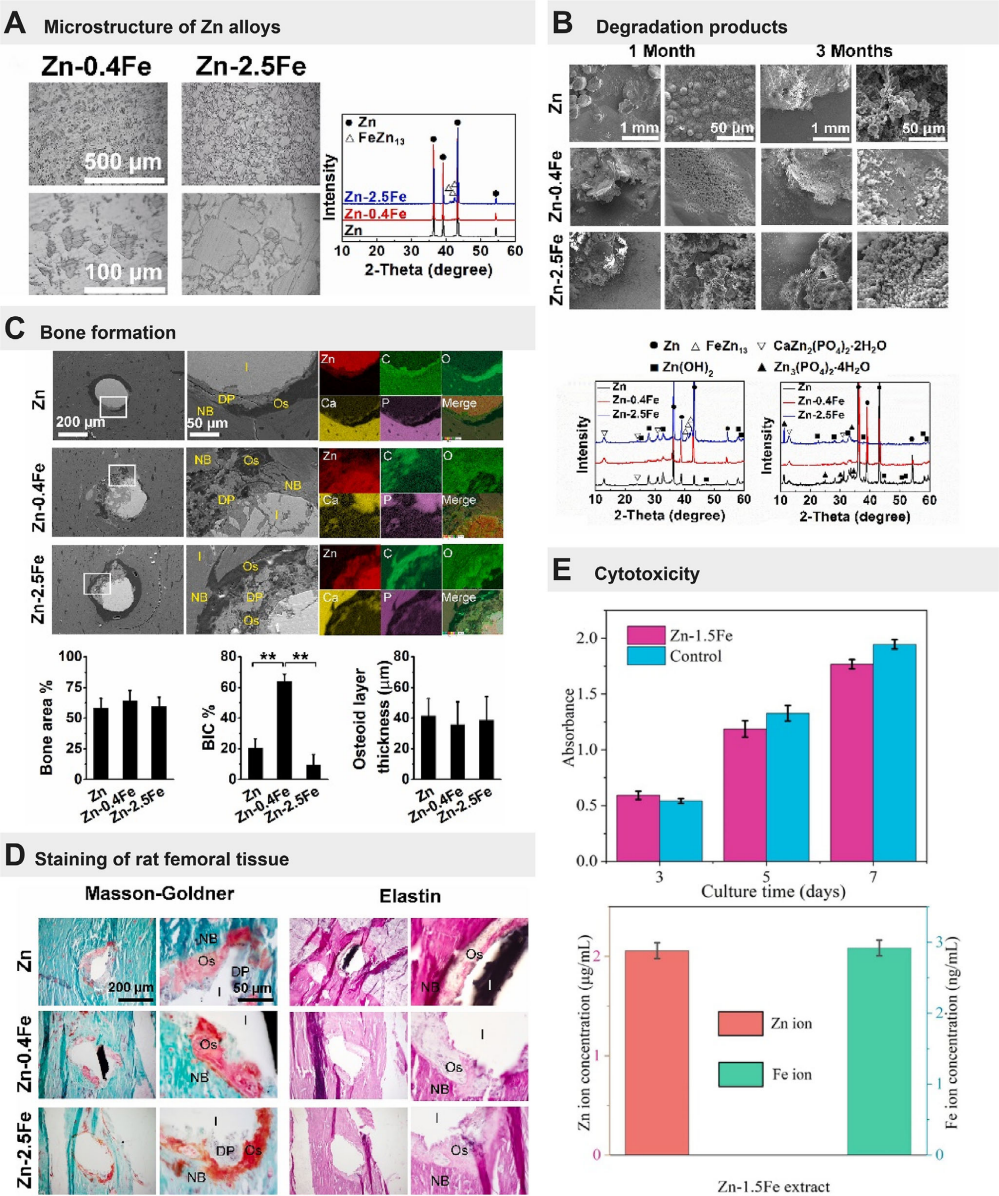
Microstructure and performance of Zn–Fe alloys. (A) Microstructures and XRD patterns of as‐extruded Zn‐Fe alloys [118]. (B) Immersion degradation of pure Zn and Zn‐Fe alloys in Hank's solution for 1 and 3 months: surface morphology and XRD patterns [118]. (C) Cross sectional SEM and EDS mapping of pure Zn and Zn‐Fe alloys after 3 months implantation in femoral tissue [118]. (D) Masson‐Goldner and Elastica van Gieson staining of rat femoral tissue with pure Zn and Zn‐Fe wire implants for 3 months [118]. Copyright 2022, Elsevier. (E) MG‐63 cell absorbance in DMEM (control) and alloy extraction medium at 3, 5, and 7 days, with Zn and Fe ion concentrations in Zn‐1.5Fe extract [147]. Copyright 2023, Elsevier.

Fe is the most abundant trace element in the human body, with approximately 4–5 g in adults [136]. It is essential for hemoglobin synthesis, electron transport, and cellular energy metabolism, contributing to local tissue repair [143, 144]. Since the solid solubility of Fe in the Zn matrix is negligible, Fe reacts with Zn to form the FeZn_13_ intermetallic compound rather than forming a solid solution [145]. Consequently, the strengthening of Zn‐Fe alloys relies primarily on second‐phase strengthening and grain refinement strengthening rather than solid‐solution strengthening [145, 146]. The bottom circulating water‐cooled (BCWC) Zn‐0.3Fe alloy comprises Zn and FeZn_13_ phases. The BCWC method achieves a cooling speed approximately 8 times that of conventional casting, which refines FeZn_13_ particles from 24 to 2 µm and Zn grains to 5 µm, eliminating detrimental lath‐like FeZn_13_ particles, reducing alloy brittleness and increasing ultimate tensile strength by 62% [145, 147]. Su et al. [118] prepared Zn‐0.4Fe and Zn‐2.5Fe alloys and evaluated their in *vitro* and in *vivo* performance. Fe addition altered alloy degradation due to differences in phase composition (Figure 17A). In Zn‐0.4Fe, fine FeZn_13_ phases supported formation of a thin passivation layer during extended degradation, whereas the dominant FeZn_13_ in Zn‐2.5Fe led to loosely accumulated degradation products (Figure 17B). Uniform degradation in Zn‐0.4Fe favored osteogenic mineralization and new bone formation, enhancing bone integration (Figure 17C,D). MG‐63 cells cultured for 7 days in Zn‐1.5Fe leachate exhibited >85% viability [147], meeting ISO 10993–5 standards [49], with Zn and Fe ion concentrations within safe limits (Figure 17E) [147].

#### 4.1.4 Zn‐Li Alloy

**FIGURE 18:**
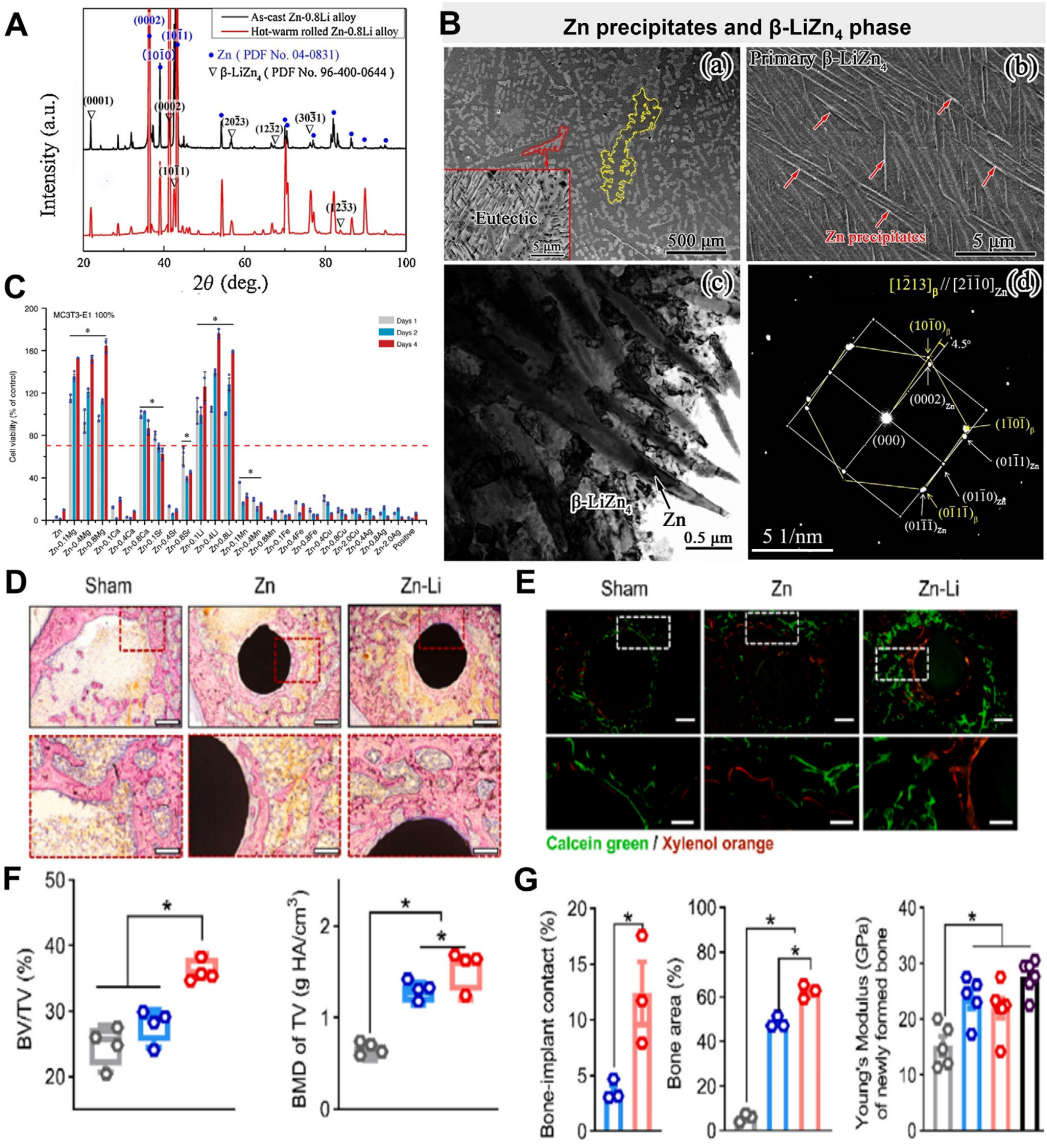
Microstructure and performance of Zn‐Li alloys. (A) XRD patterns of as‐cast and as‐rolled Zn‐0.8Li alloys [153]. (B) SEM and TEM images of the as‐cast alloy [153]. Copyright 2019, Elsevier. (C) MC3T3‐E1 cell viability cultured with alloy extracts [37]. Copyright 2020, Springer Nature. (D) Osteointegration and new bone formation at 16 weeks post‐surgery [154]. (E) Representative calcein green/xylenol orange labeling of bone regeneration around implants/defects [154]. (F) BV/TV and BMD of newly formed bone [154]. (G) Quantitative analysis of bone‐implant contact and bone area around implants/defects, and Young's modulus [154]. Copyright 2025, Elsevier.

Li is the lightest metal with a density of 0.534 g/cm^3^, reduces the density of Zn alloys when added [148, 149]. Zn–Li alloys form a Zn+α‐LiZn_4_ eutectic phase, refining the Zn grain structure [150] and producing reinforcing LiZn_4_ phases [151, 152]. This microstructure enhances mechanical properties, including tensile strength. Li et al. [153] developed an innovative process combining hot rolling at 350°C and warm rolling at 100°C to induce incomplete dynamic recrystallization, resulting in sub‐micron equiaxed Zn grains with an average size of 640 nm. This significantly enhances the matrix strength through grain refinement (Hall‐Petch relationship). Furthermore, metastable α‐Li_2_Zn_3_ nano‐precipitates with a high number density of 7.16 × 10^22^ m^−3^ and an average size of only 4.4 nm were discovered in the alloy for the first time. Combined with the high‐density needle‐like Zn precipitates formed within the β‐LiZn_4_ phase, these features exert a potent dislocation pinning effect, leading to significant precipitation strengthening. Ultimately, the alloy achieved a YS of 261.5 MPa, UTS of 401.4 MPa, and an elongation of 80.8%. Early screening of eight alloying elements (Mg, Ca, Sr, Li, Mn, Fe, Cu, Ag) showed that Li provided the strongest mechanical strengthening while improving cytocompatibility, osteogenesis, and bone integration (Figure 18C) [37]. In *vivo* studies in rat femurs demonstrated that controlled Li^+^ and Zn^2+^ release during early inflammation induces macrophage secretion of CCL5, recruiting osteogenic progenitor cells. Later, Zn^2+^ release is inhibited while Li^+^ continues anti‐inflammatory action and suppresses osteoclastogenesis, enhancing bone regeneration and implant integration (Figure 18D,E) [154]. In the Zn‐Li alloy group, the bone volume/tissue volume ratio reached 36.44% ± 1.60%, and the bone‐implant contact rate was 12.40% ± 4.87% (Figure 18F,G) [154].

#### 4.1.5 Zn‐Cu Alloy

**FIGURE 19:**
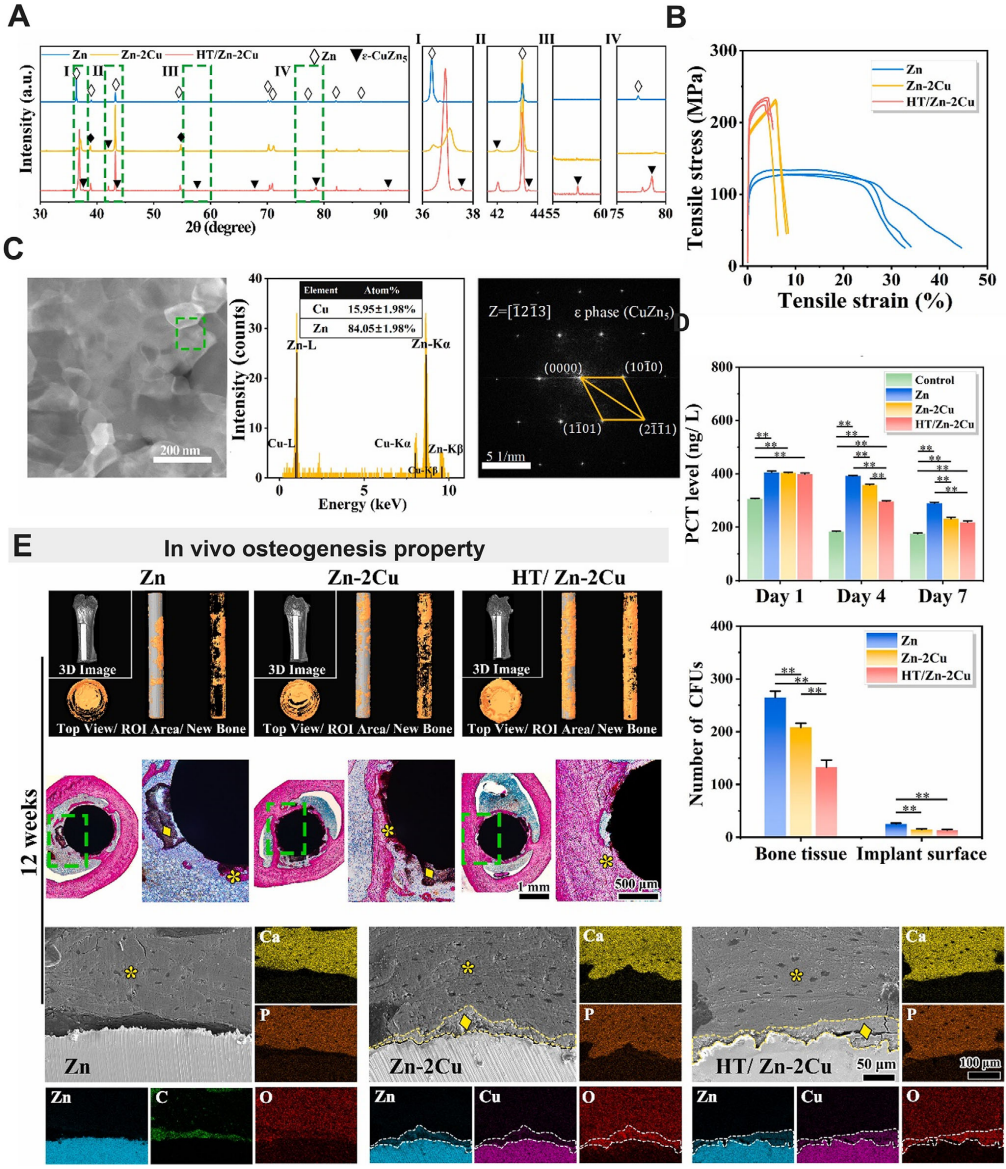
Microstructure and performance of Zn‐Cu alloys [164]. (A) XRD patterns and enlarged view containing peaks of ε‐CuZn_5._ (B) Tensile stress‐strain curves. (C) TEM, EDS, and SAED results for CuZn_5_ phase in Zn‐2Cu. (D) Serum PCT concentrations on days 1, 4, and 7 and corresponding bacterial colony counts in different rat groups. (E) In *vivo* osteogenesis property of Zn, Zn‐2Cu, and HT/Zn‐2Cu. Copyright 2026, Elsevier.

Cu is a well‐known antimicrobial element [155]. Adding 1%–5% Cu to Zn can effectively enhance the alloy's antimicrobial properties. The mechanism is primarily attributed to the contact‐killing effect of Cu^2+^, which, due to its high electrochemical potential, binds to bacterial membrane proteins [156, 157]. This interaction disrupts the integrity of the bacterial cell, leading to membrane damage and leakage of cellular contents [158, 159]. Cu has a relatively high solid solubility in Zn (maximum 2.75 wt% at the peritectic temperature), and therefore can simultaneously improve the mechanical properties of Zn through multiple strengthening mechanisms, including solid solution strengthening, second‐phase strengthening, and grain refinement strengthening [86]. When the Cu content is below the maximum solid solubility, Cu atoms dissolve into the Zn lattice to form a substitutional solid solution. These solute atoms introduce lattice distortion that impedes dislocation movement, thereby increasing the yield strength through solid solution strengthening [160, 161]. Cu additions below the peritectic composition can also act as solute providing growth restriction for the grains and the nucleation of new grains by constitutional supercooling [162]. When the Cu content exceeds the solid solubility limit, it reacts with Zn to form dendritic CuZn_5_ phases, which increase with higher Cu content. This phase formation effectively refines the grain structure of Zn, thereby improving its mechanical properties [20, 86, 163]. Liang et al. [164] used laser powder bed fusion (L‐PBF) technology to prepare pure Zn and Zn‐2Cu alloys, and combined them with a 350°C×3 h heat treatment to achieve microstructural modification. They successfully developed the HT/Zn‐2Cu alloy, which exhibits a yield strength of 202.91 ± 4.41 MPa due to the precipitation of the ε‐CuZn_5_ phase (Figure 19A–C) [164]. The combined action of Zn^2+^/Cu^2+^ through membrane disruption, ROS generation, and metal homeostasis interference provides highly efficient antibacterial properties (Figure 19D) [164], offering a new strategy for preventing and treating orthopedic infections. Concurrently, the synergistic interaction between Zn^2+^ and Cu^2+^ promotes the expression of osteogenic genes and enhances the proliferation of osteoblasts (Figure 19E) [164].

#### 4.1.6 Zn‐Ag Alloy

Zn‐Ag alloys are mainly composed of the primary η‐Zn phase together with ε‐AgZn_3_ dendrites. An increase in the AgZn_3_ volume fraction suppresses grain coarsening and leads to a finer microstructure [166, 167]. The strengthening mechanisms in Zn‐Ag alloys mainly include solid‐solution strengthening, second‐phase strengthening, and grain refinement strengthening [166]. The ε‐AgZn_3_ precipitates can effectively hinder grain boundary sliding and pin dislocations, thereby improving the strength of the alloy [168]. Meanwhile, the dissolution of Ag atoms into the η‐Zn matrix contributes to solid‐solution strengthening and enhances yield strength. Through optimized thermomechanical processing such as cold rolling and annealing, Zn‐Ag alloys can achieve an ultimate tensile strength of 432 MPa, yield strength of 385 MPa, and elongation of 34%, meeting the mechanical requirements for biodegradable implants [168]. In Zn‐2.5Ag, Zn‐5.0Ag, and Zn‐7.0Ag alloys, higher Ag contents result in a progressive rise in corrosion rate, which becomes greater than that of pure Zn. SEM observations after removing corrosion products show that samples with more AgZn_3_ secondary‐phase particles develop stronger microgalvanic corrosion, consistent with the larger Ag volume fraction [166]. At the cellular level, the expression of osteogenic genes such as Col I, BSP, and OCN, as well as proteins including ALP, Runx2, and OPN in Zn‐2Ag and Zn‐4Ag alloys exceeds that of the control group after 7–21 days of culture. In *vivo*, Zn‐4Ag implants placed in rat femoral condyles maintain direct contact with newly formed bone after 12 weeks, without fibrous tissue between the implant and host bone. Moreover, degradation sites show enriched calcium–phosphate deposition, indicating strong bone‐integration capability [169].

**FIGURE 20:**
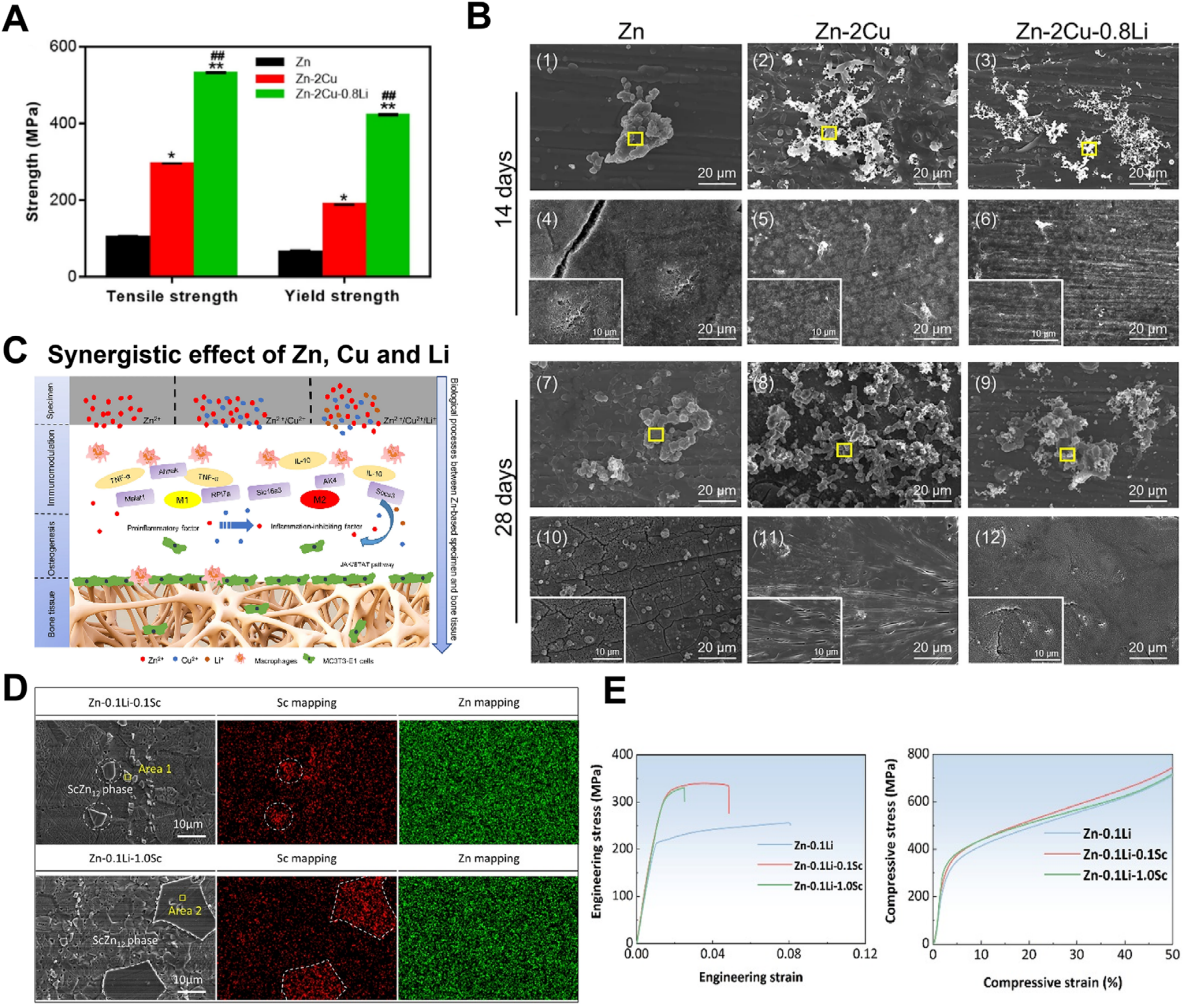
Multi‐element alloying strategies for Zn‐based systems. (A) Tensile and yield strengths of pure Zn, Zn‐2Cu, and Zn‐2Cu‐0.8Li. (B) SEM images of the corroded Zn, Zn‐2Cu, and Zn‐2Cu‐0.8Li samples. (C) Biological processes between Zn‐based specimen and bone tissue [121]. Copyright 2025, Springer. (D) Typical ScZn_12_ phases observed in Sc‐containing alloys and their corresponding elemental distributions. (E) Tensile and compressive stress‐strain curves of Zn‐Li‐based alloys [165]. Copyright 2025, Elsevier.

The addition of the rare‐earth element Dy (Zn‐5Dy) enhances bioactivity and osseointegration by modulating mitochondrial responses through SIRT4‐dependent regulation [170]. As research on Zn alloys expands, alloy design has shifted from single‐element additions to multicomponent strategies aimed at improving overall performance. With Li as the sole alloying element, Zn‐2Li exhibits a UTS of 360 MPa, which further increases to 420 MPa in Zn‐4Li [121], representing a two‐ to four‐fold enhancement over pure Zn. When Li is combined with Cu, the Zn‐2Cu‐0.8Li alloy reaches a UTS of 517.33 MPa and a YS of 422.82 MPa while maintaining a relatively low corrosion rate (Figure 20A,B) [121]. Both Zn‐2Cu and Zn‐2Cu‐0.8Li alloys drive macrophage polarization toward the anti‐inflammatory M2 phenotype through the co‐release of Cu^2+^ and Li^+^ ions, thereby reducing pro‐inflammatory mediators such as TNF‐α and promoting osteogenic differentiation of MC3T3‐E1 cells (Figure 20C) [121, 125]. Huang et al. [165] introduced Sc into the Zn‐0.1Li alloy, generating the ScZn_12_ phase (Figure 20D) and further refining the microstructure. This modification increased the ultimate tensile strength from 257 MPa to 341 MPa (Figure 20E). Although galvanic coupling between Sc and the Zn matrix accelerated corrosion, the alloy maintained excellent biocompatibility. In the Zn‐0.45Mn‐0.2Mg alloy, grain refinement and the presence of Mg_2_Zn_11_ and MnZn_13_ second phases yielded mechanical properties suitable for orthopedic applications. The alloy displayed antibacterial activities of 96.2% against *E. coli* and 97.3% against *S. aureus*, while Mg^2+^ release mitigated cytotoxicity and supported osteogenic responses [171]. Chen et al. [172] examined the aging hardening behavior and microstructural evolution of the Zn‐1.5Cu‐1.5Ag alloy under different thermal treatments. They found that aging at 150°C for 48 h stabilized mechanical performance, offering a suitable post‐processing strategy for vascular stent applications.

### 4.2 Processing Routes

#### 4.2.1 Rolling

Rolling, which includes hot rolling (HR), cold rolling (CR), and multi‐step thermomechanical processes, is one of the most widely used methods in zinc alloy processing and is suitable for sheet metal, plates, and foil materials [173]. Hot rolling, typically conducted between 100°C and 300°C, induces dynamic recrystallization (DRX) that transforms coarse as‐cast dendritic structures into fine equiaxed grains while fragmenting continuous intermetallic networks into finely dispersed discrete particles [119]. Tong et al. [119] demonstrated that hot rolling of Zn‐5Ge alloys markedly refined the eutectic Ge phase and homogenized its distribution, achieving a UTS of 237.0 MPa, substantially higher than that of as‐cast pure Zn. Chen et al. [174] found that two‐step hot rolling exerted a remarkable modifying effect on Zn‐0.8Mg alloys, boosting elongation from merely 1.4% after the first‐step hot rolling at 300°C to 21.3% after secondary rolling at 200°C, mainly owing to sufficient dynamic recrystallization, ultrafine grain refinement in eutectic regions, and uniform dispersion of MgZn_2_ and Mg_2_Zn_11_ precipitates. Li et al. [175] proved that hot rolling effectively overcame the intrinsic brittleness of as‐cast Zn‐Cu alloys, raising the ultimate tensile strength of Zn‐4Cu alloy to 393.3 MPa and maintaining elongation above 38%, with particle‐stimulated nucleation during rolling accelerating dynamic recrystallization and refining matrix grains. Cold rolling introduces intense strain hardening that further lifts the strength of Zn alloys, yet excessive deformation tends to cause embrittlement and degraded ductility. Hybrid rolling routes integrating hot rolling, cold rolling, and intermediate tempering have been proven effective in balancing strength and ductility. For example, Cui et al. [176] found that room‐temperature cold rolling endowed Zn‐2Cu alloy with higher yield strength, but elongation dropped to 23.4%, far lower than that of the multi‐pass hot‐rolled state.

#### 4.2.2 Extrusion

Hot extrusion is the most extensively utilized processing route for biodegradable Zn alloys, particularly for rod‐shaped and tubular products intended for orthopedic fixation devices and vascular stents [177]. The high shear strains generated during extrusion effectively break up coarse intermetallic compounds and redistribute them as fine, homogeneously dispersed particles within the Zn matrix, which significantly mitigates the deleterious effects of continuous brittle networks on ductility [178].

Extruded Zn‐Mg‐Sr alloys achieve excellent strength‐ductility balance, with a UTS of 324 MPa and elongation up to 20% [179]; Čapek et al. [179] demonstrated that this superior performance of extruded Zn‐0.8Mg‐0.2Sr alloy originated from fully recrystallized fine equiaxed grains and uniform dispersion of Mg_2_Zn_11_ and SrZn_13_ precipitates, and Klíma et al. [180] further verified its outstanding in *vivo* biocompatibility and controllable degradation in 1 year rabbit tibia implantation model. For Zn‐Cu alloys, Zhang et al. [181] reported that hot‐extruded Zn‐0.5Cu‐0.2Fe alloy exhibited a UTS of 202.3 MPa and exceptional elongation of 41.2% for guided bone regeneration applications, an effect attributed to extrusion‐induced grain refinement and homogeneous FeZn_13_ particle distribution, which also accelerated degradation and endowed effective antibacterial properties. The extrusion parameters significantly influence the final microstructure and properties. Higher extrusion ratios generally produce finer grains and superior mechanical properties but may introduce surface defects. Indirect (backward) extrusion, which reduces friction between the billet and container, has been shown to produce more homogeneous microstructures compared to direct extrusion [182].

#### 4.2.3 Additive Manufacturing

Additive manufacturing (AM), particularly laser powder bed fusion (LPBF), has emerged as a highly promising fabrication technique for biodegradable Zn implants in recent years. AM offers unique advantages, including the ability to fabricate patient‐specific complex geometries, precisely controlled porous architectures for bone tissue engineering scaffolds, and near‐net‐shape manufacturing that minimizes material waste [183]. The rapid solidification inherent to LPBF (cooling rates of 10^3^–10^6^ K/s) produces ultrafine microstructures with significantly refined grains and extended solid solubility, which can enhance both mechanical properties and corrosion uniformity [184].

Dong et al. [185] systematically investigated the anisotropic mechanical and degradation properties of LPBF‐fabricated pure Zn, revealing that its horizontal plane achieved a UTS of 123.6 MPa, the vertical plane reached an elongation of 14.2%, and clarified the underlying deformation mechanisms while verifying its favorable osteogenic potential. Qin et al. [186]fabricated novel Zn‐xWE43 porous scaffolds via LPBF, achieving high densification over 99.47% for bulk samples, and demonstrated that the addition of WE43 induced remarkable grain refinement and second phase precipitation; the optimized Zn‐5WE43 bulk alloy exhibited the highest tensile strength of 335.4 MPa, and its corresponding porous scaffold delivered a compressive strength of 73.2 MPa and Young's modulus of 2480 MPa, while the massive formation of brittle MgZn_2_ phase in Zn‐8WE43 significantly deteriorated its mechanical performance. Shuai et al. [187] optimized LPBF processing to fabricate Zn‐2Al alloy with a high densification rate of 98.3%, which exhibited balanced mechanical properties, a moderate degradation rate matching bone healing, and good in *vitro* cytocompatibility. Qin et al. [188] systematically evaluated LPBF‐fabricated Zn‐xMg porous scaffolds, demonstrating that the Zn‐1Mg scaffold possessed mechanical properties highly compatible with human cancellous bone, and in *vivo* studies confirmed its enhanced osseointegration and excellent biocompatibility.

However, AM of Zn alloys faces several unique challenges. The low boiling point of Zn (907°C) relative to its melting point (420°C) leads to significant metal evaporation, fume generation, and consequent porosity during laser processing [189, 190]. The high vapor pressure of Zn also causes spatter formation and potential loss of volatile alloying elements, which can result in compositional deviation from the designed alloy.

In addition to the above‐mentioned primary processing routes, severe plastic deformation (SPD) techniques have been investigated to fabricate ultrafine‐grained Zn alloys with exceptional properties. Equal channel angular pressing (ECAP) refines grains to the sub‐micrometer regime, delivering significant strengthening via the Hall–Petch mechanism [191]; Saksl et al. [191] reported that two‐step ECAP‐processed Zn‐0.1Mg alloy was refined to an ultrafine‐grained structure of 0.5–1.0 µm, with exceptional room‐temperature superplasticity and simultaneous significant improvement in both strength and ductility. High‐pressure torsion (HPT) can produce ultrafine‐grained structures in dual‐phase Zn alloys, but generally results in coarser grains than ECAP in low‐alloyed quasi‐single‐phase Zn systems due to dynamic recrystallization and grain growth [192].

### 4.3 Surface Modification

**FIGURE 21:**
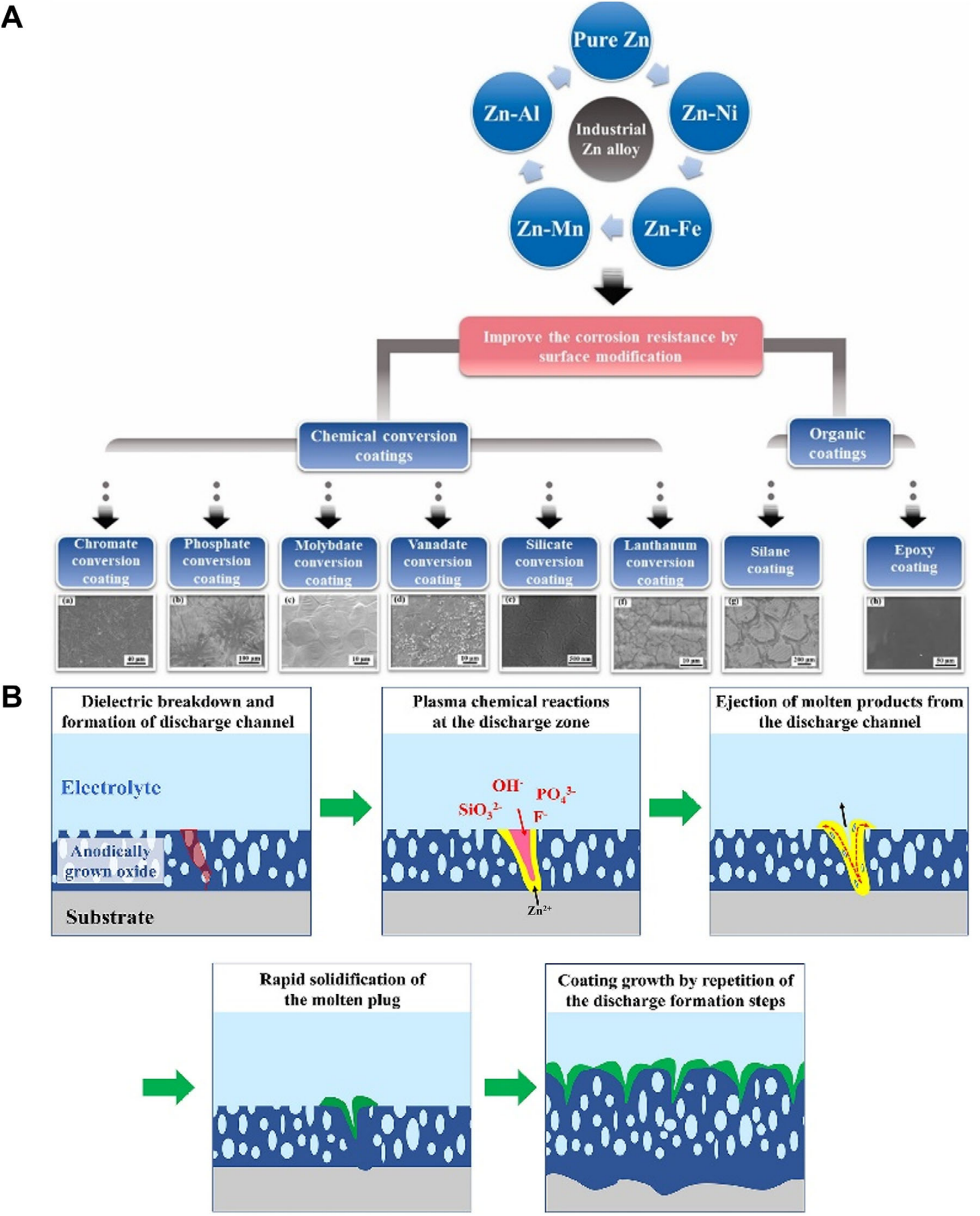
Representative surface modification techniques for Zn alloys. (A) Representative strategies for surface modification of industrial Zn‐based alloys [194]. Copyright 2022, Elsevier. (B) Schematically illustrating MAO coating formation process [195]. Copyright 2022, Elsevier.

Surface modification is also an effective way to modify alloys. It is a technique used to improve surface properties, impart new surface functions, and develop implant materials with appropriate mechanical properties [193]. Surface modification can optimize degradation behavior and bioactivity without changing the properties of the substrate. Common surface modification methods include micro‐arc oxidation, sandblasting, anodizing, and hydrothermal methods (Figure 21A) [194].

**FIGURE 22:**
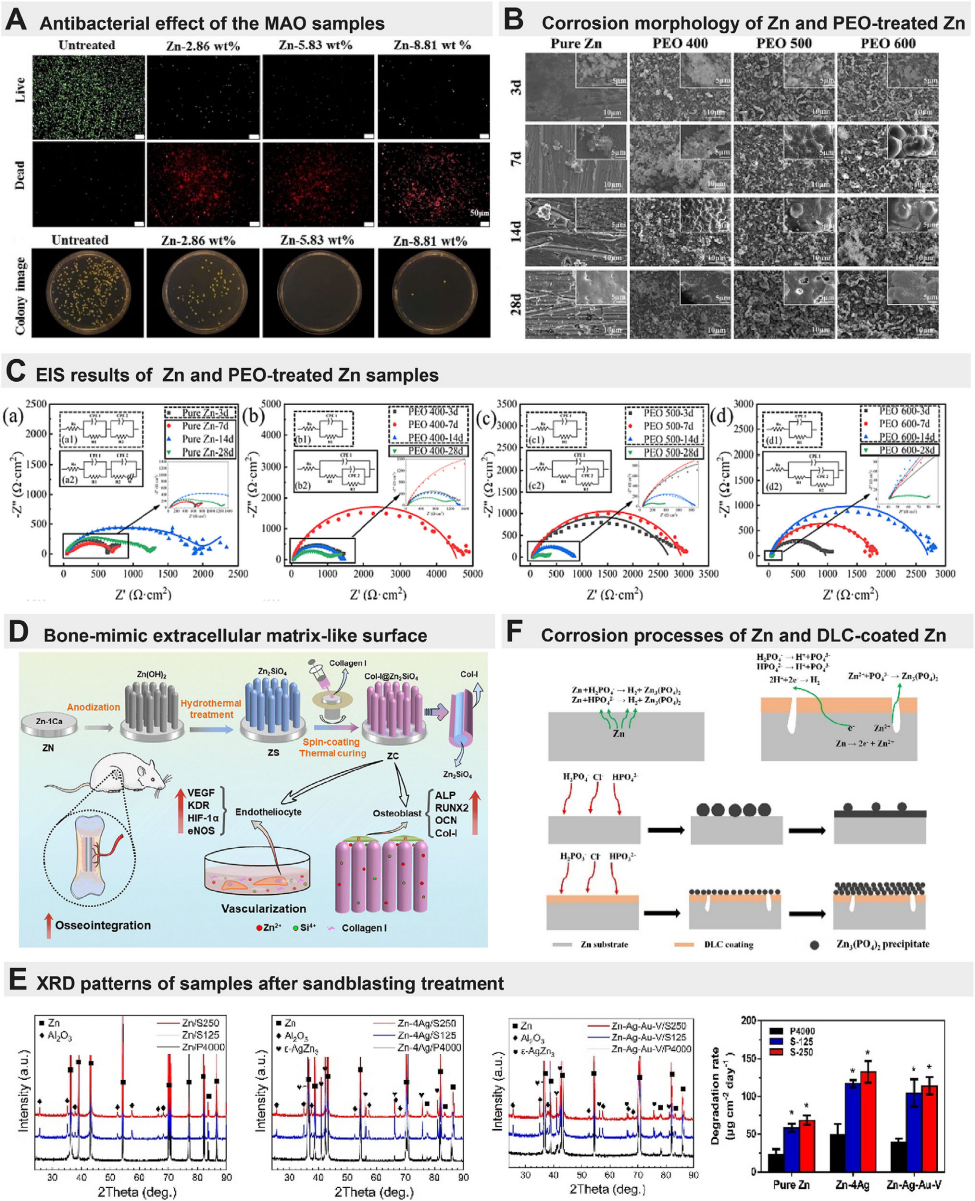
Corrosion protection and antibacterial coatings for Zn alloys. (A) Antibacterial performance of MAO‐treated samples after 24 h of incubation with *S. aureus* [197]. Copyright 2023, Elsevier. (B) SEM images of pure Zn and PEO‐treated Zn after immersion in SBF for 3, 7, 14, and 28 days [200]. (C) EIS responses of untreated and PEO‐treated Zn samples after immersion in Hank's solution for 3–28 days [200]. Copyright 2023, Elsevier. (D) Fabrication process of a bone‐mimicking extracellular‐matrix‐like surface on Zn‐1Ca alloy [201]. Copyright 2022, American Chemical Society. (E) XRD patterns and degradation rates of pure Zn, Zn‐4Ag alloy, and Zn‐Ag‐Au‐V alloy [203]. Copyright 2023, Elsevier. (F) Corrosion reactions and layer‐formation mechanisms in bare Zn and DLC‐coated Zn [204]. Copyright 2021, American Chemical Society.

Micro‐arc oxidation (MAO), also known as plasma electrolytic oxidation (PEO), is a unique process developed based on the traditional anodizing reaction. It typically uses metals such as Mg, Al, Ti, or their alloys as the anode [196]. During the process, high pressure generated instantaneously in the electrolyte is used to break down the metal surface. The substrate metal surface undergoes a series of chemical transformations, resulting in the formation of a ceramic oxide layer (Figure 21B) [195]. Liu et al. [197] studied the long‐term antibacterial performance and mechanism of a Zn‐doped MAO coating on Ti6Al4V. They found that the Zn‐5.83 wt.% coating exhibited an antibacterial rate of 100% against *S. aureus* initially. However, after 14 days of immersion, the antibacterial rate dropped to 12.5%, indicating that the coating's antibacterial capability was strong in the early stages but decreased over time (Figure 22A) [197]. Shi et al. [198] applied hydrothermal treatment (HT) combined with MAO to pure Zn substrates to fabricate a CaZn_2_(PO_4_)_2_·2H_2_O (CaZnP) coating. This treatment enhanced the biocompatibility of pure Zn and regulated its degradation rate. Zhu et al. [199] developed a bioactive ceramic composite coating on Zn‐Mn‐Mg alloys using a combined micro‐arc oxidation and hydrothermal (MAO‐HT) process. The MAO‐HT‐5 h coating showed increased hydrophilicity and markedly enhanced the viability of MC3T3‐E1 osteoblasts and L‐929 fibroblasts. Chen et al. [200] investigated the in situ formation of a ZnO/Zn(OH)_2_ hybrid layer on pure Zn via plasma electrolytic oxidation (PEO) in a Na_2_CO_3_‐based electrolyte. Following PEO treatment, both in *vitro* biocompatibility and antibacterial activity improved substantially. At an applied voltage of 400 V, the initial degradation rate rose from that of untreated Zn to 0.60 mm/year, while the long‐term corrosion rate stabilized between 0.50–0.70 mm/year, enabling controlled degradation behavior (Figures 22B,C).

Anodizing is widely used to generate a stable, uniform, and sufficiently thick oxide layer on anode surfaces [193]. Mao et al. [201] created bone‐like extracellular matrix (ECM) surfaces on Zn‐1Ca implants through a combined process of anodic oxidation, hydrothermal treatment, and fluorine curing (Figure 22D). This hybrid modification markedly enhanced osteoblast adhesion, proliferation, and differentiation on the implant surface, and also promoted endothelial cell vascularization in the extract medium. Guillory et al. [202] treated pure Zn samples with electro polishing (EP) and anodizing (AD), designing a unique oxide film. They found that the samples treated with AD exhibited better corrosion resistance.

Sandblasting typically uses compressed air as a power source to form a high‐speed jet that propels abrasive particles onto the surface of the sample, thereby altering its roughness. The process is simple and easy to operate [205]. Li et al. [203] found that sandblasting treatment on pure Zn increased the degradation rate, and that increasing the sandblasting particle size from 125 to 250 µm did not significantly affect the degradation rate (Figure 22E) [203].

In addition, Xiang et al. [85] applied a one‐step surface acid etching treatment using 5% and 15% nitric acid. By increasing the surface roughness and Zn^2+^ release, they significantly inhibited the biofilm formation of *S. aureus*. The surface of Zn treated with 15% nitric acid exhibited a needle/leaf‐like nanocrystalline structure, showing better antibacterial effects compared to the 5% acid‐etched group. Peng et al. [204] deposited a diamond‐like carbon (DLC) coating on the Zn surface using magnetron sputtering and found that the DLC coating accelerated the corrosion of the substrate, likely due to the occurrence of galvanic corrosion between the substrate and the coating (Figure 22F) [204].

### 4.4 Surface Functionalization

Surface engineering represents a pivotal strategy to bridge the gap between Zn‐based implants and physiological environments. To further optimize the bone‐implant interface, bio‐inspired strategies derived from natural hierarchical materials offer promising insights [206]. For instance, the multi‐level organization and unique balance of strength and elasticity found in the insect cuticle have inspired the development of sophisticated biomimetic protective coatings and functional interfaces [207]. Such bionic architectures can be leveraged to modulate the degradation kinetics of Zn alloys while simultaneously enhancing biological integration. Building upon these natural archetypes, researchers have developed various specialized coatings, including nanostructured surfaces and drug‐eluting layers. These surface designs not only boost biological activity but also enable targeted therapeutic effects, supporting bone repair and reducing infection risks.

**FIGURE 23:**
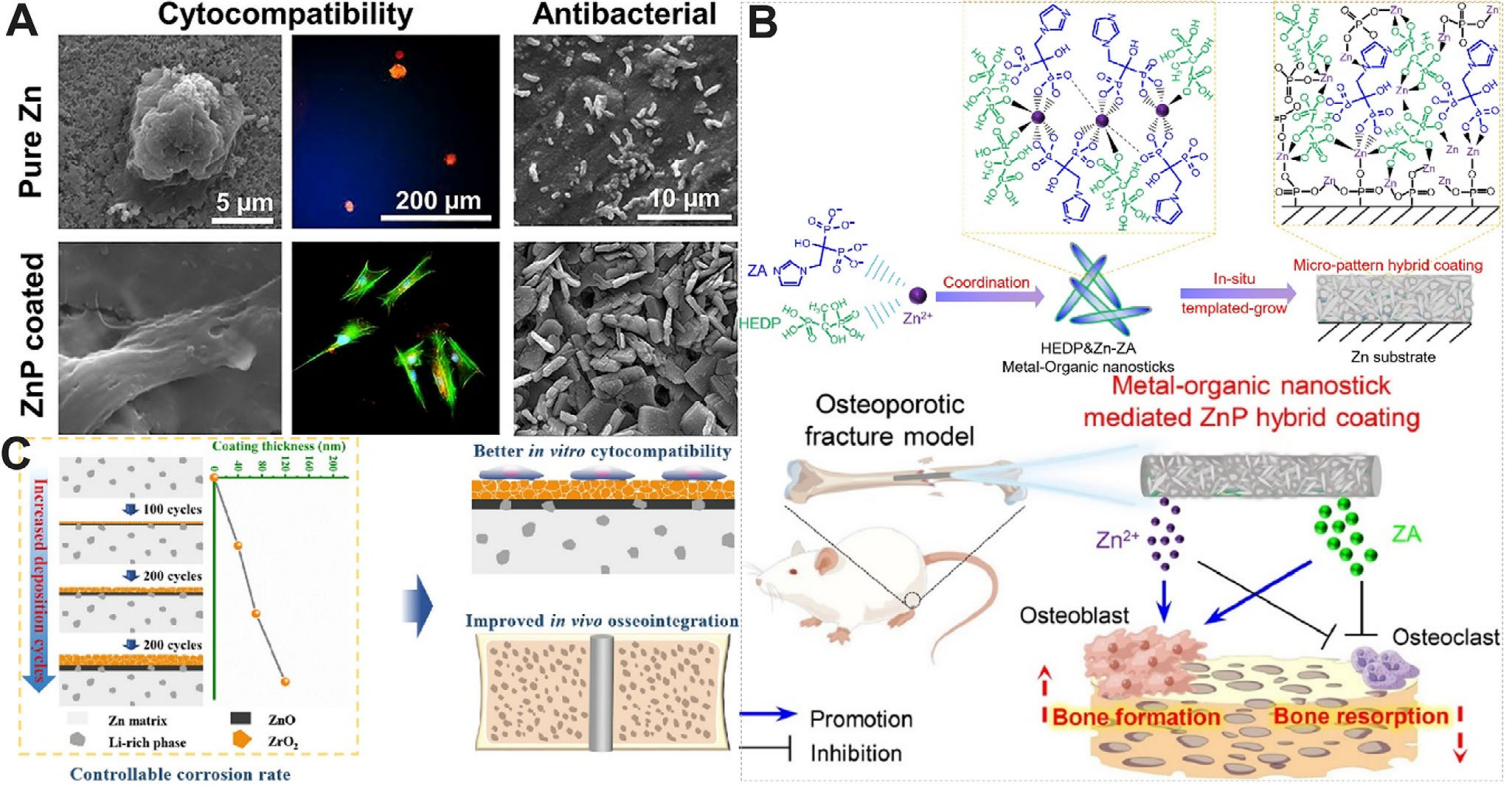
Functional surface chemistries for Zn‐based implants. (A) Cytocompatibility and antibacterial activity of ZnP‐coated Zn versus pure Zn [208]. Copyright 2019, Elsevier. (B) Schematic of a Zn‐phosphate hybrid layer formed via metal‐organic nanorods that promotes osteoporotic fracture healing by enhancing bone formation and suppressing osteoclast resorption [210]. Copyright 2023, Elsevier. (C)ZrO_2_ atomic layer deposition and its effects on cytocompatibility and osseointegration [209]. Copyright 2020, Elsevier.

To address the inherent challenge of rapid initial degradation and uncontrolled Zn^2+^ burst release, inorganic and composite barrier coatings have been widely investigated as primary interfacial regulators. Chemical conversion processes offer a straightforward approach; for instance, Su et al. [208] prepared a micro/nano‐scale zinc phosphate (ZnP) coating that acts as a sacrificial layer to moderate degradation while improving initial antibacterial behavior(Figure 23A). For applications requiring higher structural precision, atomic layer deposition (ALD) provides superior topological control. Yuan et al. [209] used atomic layer deposition to form a ZrO_2_ nanofilm on Zn‐Li alloys, enabling precise control of degradation and significantly enhancing both cytocompatibility and osseointegration (Figure 23C) [209]. To further bridge mechanical protection with biological affinity, composite strategies are employed. Qian et al. [210] developed a wiener‐structured metal‐organic/inorganic hybrid layer (HEDP & Zn‐ZA/ZnP), which effectively moderated Zn^2+^ release and met the dual demands of corrosion control and osteoporosis repair (Figure 23B). Shoeib et al. [211] fabricated a nano‐hydroxyapatite/chitosan coating using a microwave method, the coating showed high crystallinity, strong corrosion resistance, and balanced osteogenic and antibacterial functions. Owing to their micro/nanoscale features, these coatings help regulate Zn^2+^ release and degradation while improving biocompatibility, bone integration, and corrosion protection, thus supporting the safe and effective use of Zn‐based implants in clinical applications.

**FIGURE 24:**
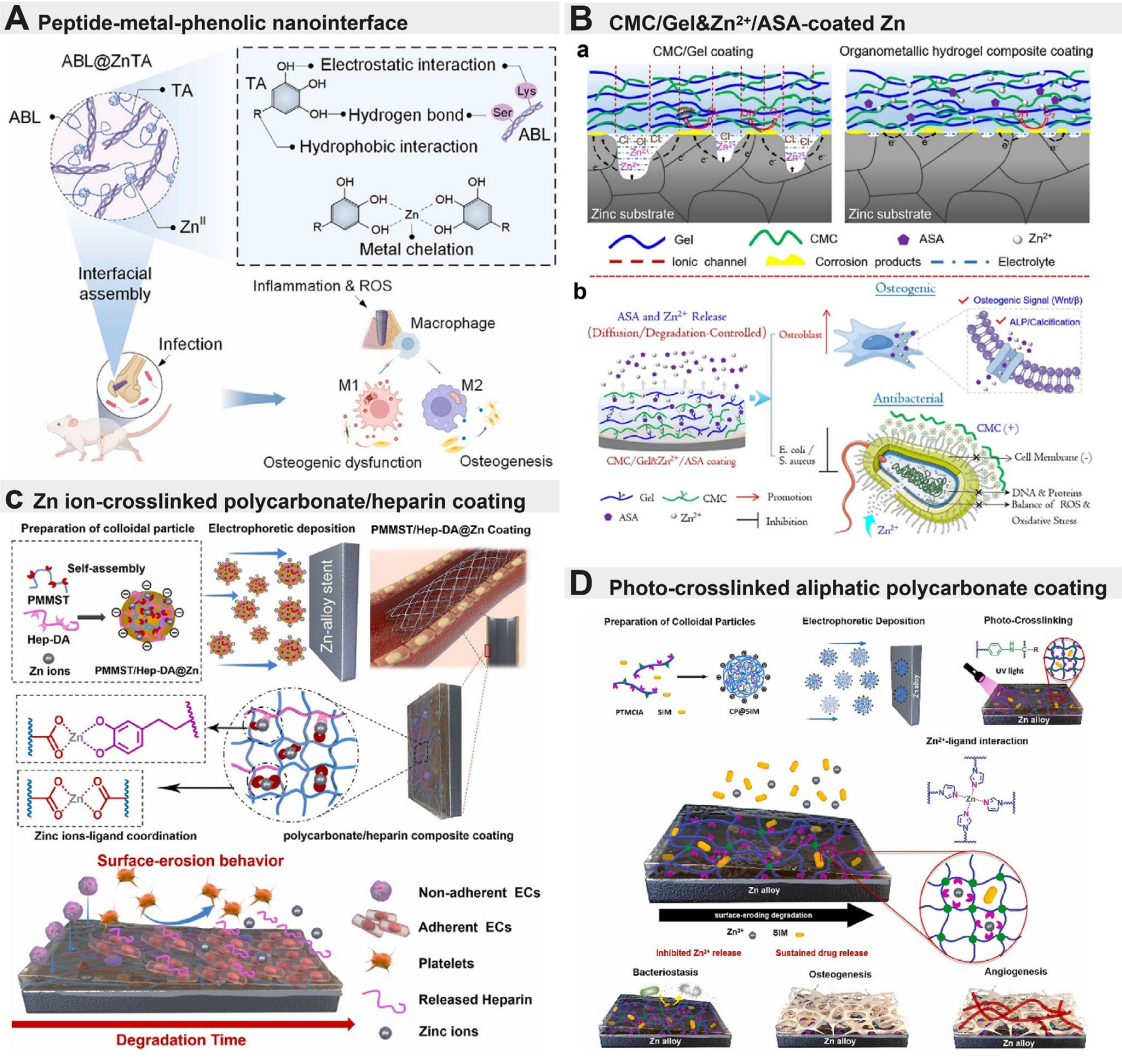
Biofunctional coatings for controlled degradation and regeneration. (A) A peptide‐metal‐phenolic nanointerface for bone‐implant integration [212]. Copyright 2024, Elsevier.(B‐a) Corrosion‐protection mechanism of CMC/Gel&Zn^2+^/ASA‐coated Zn versus CMC/Gel‐coated Zn. (b) Zn^2+^ and ASA release pathways and resulting osteogenic and antibacterial effects [213]. Copyright 2023, Elsevier. (C) Zn‐ion‐cross‐linked polycarbonate/heparin coating on Zn‐alloy stents with surface‐erosion behavior via electrophoretic deposition [214]. Copyright 2022, Elsevier. (D) Photo‐cross‐linked polycarbonate drug‐eluting coating on Zn alloys, showing degradation, drug release, and associated osteogenic, angiogenic, and antibacterial effects [215]. Copyright 2024, Elsevier.

In addition, drug‐loaded coatings containing anti‐osteoporosis agents, anti‐inflammatory molecules, or antibacterial drugs can provide Zn implants with targeted therapeutic functions. Such coatings enable controlled Zn^2+^ release and tuned degradation while delivering synergistic benefits, including corrosion regulation and enhanced biological activity, to meet diverse clinical needs. Xu et al. [212] designed a Zn finger‐inspired peptide‐metal‐phenolic nanointerface (ABL@ZnTA) by integrating the osteoporotic drug abaloparatide (ABL) into a zinc‐tannic acid (Zn‐TA) coating on Ti implants. Although initially validated on Ti implants, this robust Zn‐tannic acid coordination system provides a universal strategy that maybe adapted to Zn alloys, significantly multiplying implant‐bone bonding strength and functional connectivity. This approach achieved a 1.36‐fold higher drug loading capacity and improved the implant‐bone bonding strength by 6 times and functional connectivity by 1.5‐3 times in a rat bone defect model (Figure 24A) [212]. Qian [213] developed a composite coating of carboxymethyl chitosan (CMC)/gelatin (Gel)‐Zn^2+^ organic metal hydrogel loaded with aspirin (ASA) (CMC/Gel&Zn^2^^+^/ASA). This coating was constructed on the Zn surface using an alternating dipping method, protecting the Zn substrate from localized pitting corrosion (Figure 24B‐a) [213]. It demonstrated good antibacterial properties, with new bone formation on the implant surface. This approach successfully achieved the synergistic optimization of corrosion control, osteogenesis, anti‐inflammation, and antibacterial effects on Zn‐based implant(Figure 24B(b)) [213]. A Zn ion‐cross‐linked polycarbonate/heparin composite coating (PMMST/Hep‐DA@Zn) was fabricated via electrophoretic deposition (EPD). This coating offers an effective surface‐modification strategy for biodegradable cardiovascular Zn‐based implants, providing anti‐corrosion, anticoagulant, and endothelialization‐promoting properties(Figure 24C) [214]. Zhang et al. [215] developed a biodegradable aliphatic polycarbonate drug‐eluting coating (Zn‐CP@SIM) on the surface of Zn‐Li alloy. The coating was formed by EPD and UV cross‐linking of aliphatic polycarbonate (PTMCIA) and simvastatin (SIM). This coating exhibited surface erosion characteristics and incorporated imidazole side groups to inhibit Zn^2+^ release through Zn^2+^‐imidazole coordination. It also utilized the osteogenic, pro‐angiogenic, and antibacterial properties of SIM, achieving long‐term corrosion resistance, multifunctional bioactivity, and excellent bone repair performance for Zn alloys (Figure 24D) [215].

Surface functionalization has evolved from providing simple physical barriers to constructing dynamic, multifunctional interfaces that address the complex physiological demands of Zn‐based implants. Despite these significant advancements, critical challenges remain for clinical translation. The dynamic nature of the degrading Zn substrate often compromises the long‐term interfacial adhesion of the coatings, potentially leading to premature delamination under cyclic physiological loading. Furthermore, achieving uniform and stable coating distribution on complex geometries (such as porous bone scaffolds) remains technically demanding.

## 5 Pathways toward Clinical Translation

### 5.1 In *Vitro* and In *Vivo* Degradation Evaluation

There are two main methods for testing the in *vitro* degradation of implants: one is the immersion experiment, and the other is electrochemical testing. In the immersion experiment, the sample to be tested is immersed in the corresponding solution for a specific period. Different artificial solutions mimicking the physiological environment, such as PBS, Hank's solution, Ringer's solution, revised simulated body fluid (r‐SBF), and human plasma have been used for in *vitro* tests [60]. After immersion, the surface morphology before and after removing the corrosion products can be observed using techniques such as SEM and laser confocal microscopy. The corrosion products can then be analyzed, and the corrosion rate can be calculated. A widely used standard for immersion testing is ASTM G31‐72 [73]. The corrosion rate can be evaluated according to the mass loss of the sample, according to the equation (18) [216].

(18) R=W/Atρ

R—corrosion rate, W—weight loss, A—the area exposed to corrosion of the initial sample, t—exposure time, and ρ—density of exposed alloy.

Electrochemical testing is another important method used in in *vitro* degradation studies. Typically, a three‐electrode workstation (working electrode, reference electrode, and counter electrode) is employed. This method mainly involves measuring the open circuit potential (OCP) and electrochemical impedance spectroscopy (EIS) to obtain Nyquist and Bode plots. Polarization curves are recorded, and the corrosion potential (E_corr_) and corrosion current density (i_corr_) of different samples are determined using the Tafel extrapolation method [185]. ISO 16429:2004 provides detailed and specific guidelines for this testing method, outlining the procedures and requirements for conducting electrochemical degradation studies of materials used in medical devices and implants [217]. Using the corrosion current density, the polarization resistance and the corrosion rate can be determined according to relations (19) and (20) [216].

(19) $$R_{p}=\frac{\beta_{a}*\beta_{c}}{2.3\left(\beta_{a}+\beta_{c}\right)*i_{\mathrm{corr}}}$$

(20) $$E_{\mathrm{corr}}=\frac{i_{\mathrm{corr}}*A}{\rho *z*F}\;*NS_{\mathrm{year}}$$

*R_p_*—polarization resistance, [Ω]; βa×βc—anodic and cathodic Tafel slopes, [mV/dec]; i_corr_—corrosion current density, [mA/cm^2^]; *E_corr_*—corrosion rate, [mm/year]; A—atomic mass, [g]; ρ—density, [g / cm^3^]; z – valence; F—Faraday's constant, [C]; NS_year_—number of seconds in a year = 3.1536 × 10^7^.

Assessment of in *vivo* degradation is a key step in verifying the biosafety and degradation behavior of biodegradable biomaterials, and this process relies primarily on animal models (such as rat femoral condyle defects or rabbit radial defects). Before conducting animal experiments, the experimental protocol must be submitted to the relevant ethics committee for approval. Only after receiving approval can the next steps be carried out. After selecting the appropriate animal model, the implant is introduced into the animal's body. After a fixed postoperative period (e.g., 4 weeks, 8 weeks, 12 weeks [140]), the animal is euthanized, and the surgical site is subjected to micro‐computed tomography (micro‐CT). SEM equipped with EDS is used to observe the surface morphology and elemental composition of the Zn alloy after surgery, as well as the surface morphology after removal of the degradation products [218]. By analyzing the morphology and elemental composition of the degradation products, the main components and corrosion mechanisms of the degradation products can be determined. Before and after the surgery, the mass/volume of the implants should be measured, and the corrosion rate calculated to evaluate and analyze the in *vivo* degradation process.

### 5.2 Preclinical Animal Studies

Before clinical application, animal experiments (involving mice, rats, dogs, rabbits, etc.) are an essential step. Only when the implants demonstrate good performance in animal studies can they potentially be suitable for clinical use. Animal studies need to select different animal types and implantation sites based on the type of implant. Before implantation, in *vitro* biological tests such as live/dead cell staining and CCK8 assays should be performed to evaluate the biocompatibility of the material before proceeding to animal trials. After implantation, in addition to analyzing the degradation behavior of the implant, other research work is required: histological analysis (HE staining, Masson trichrome staining [118, 219]) to evaluate local inflammation, new bone formation (bone volume fraction BV/TV [220]), and elemental distribution detection (fluorescent staining, X‐ray energy spectroscopy [221]) to clarify the metabolic pathway of Zn^2+^ in bone tissue, avoiding ion accumulation in distant organs.

**FIGURE 25:**
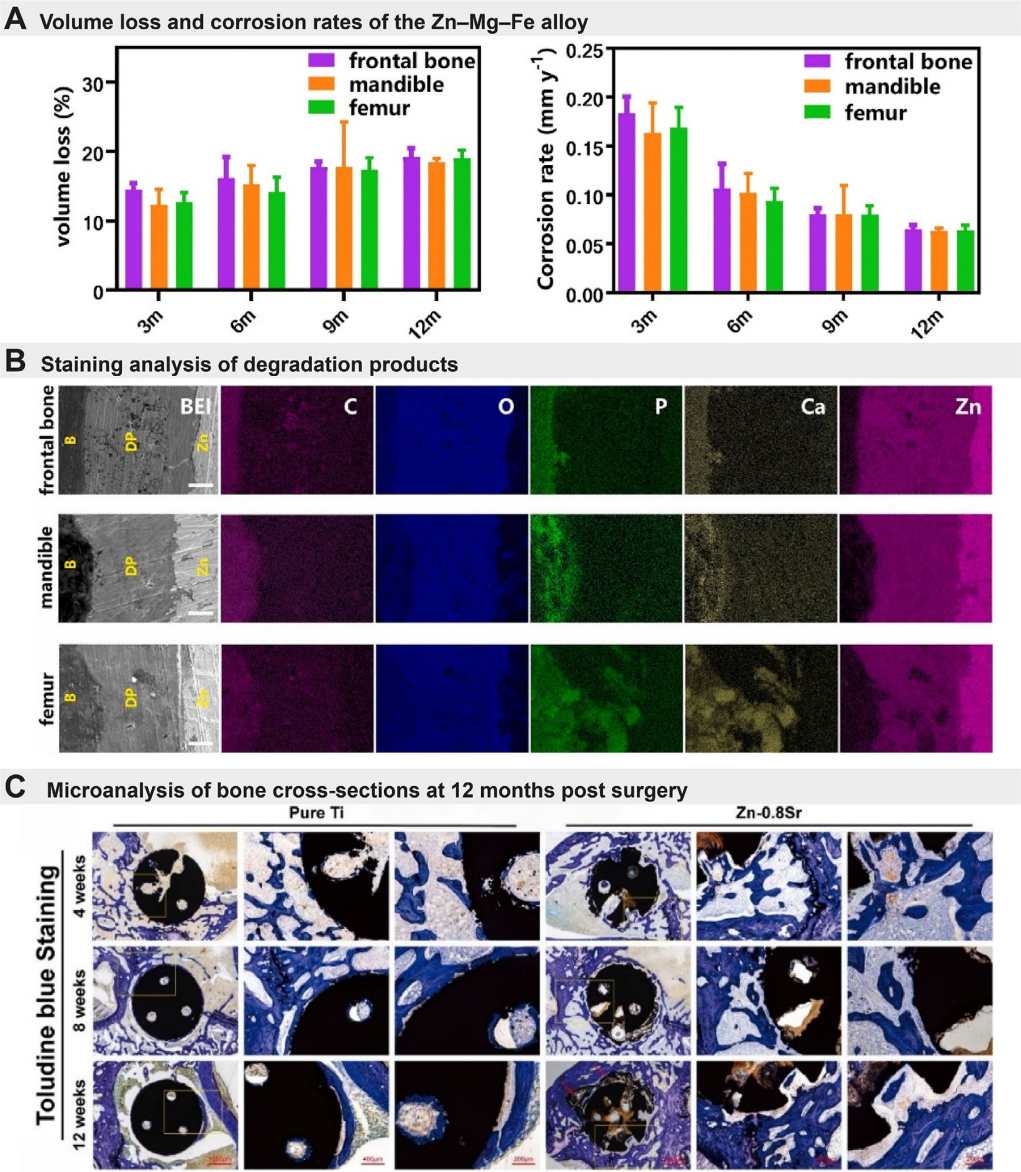
In vivo degradation and tissue integration of Zn‐based implants. (A) Volume loss and corrosion rates of Zn‐Mg‐Fe alloy implanted in the frontal bone, mandible, and femur at 3, 6, 9, and 12 months post‐surgery [55]. (B) SEM images of frontal bone, mandible, and femur cross sections 12 months post‐implantation, with corresponding elemental mapping [55]. Copyright 2022 Elsevier. (C)Toluidine blue staining of pure Ti and Zn‐0.8Sr alloy scaffolds, showing biodegradable products of Zn‐0.8Sr 12 weeks after implantation (red arrows) [222]. Copyright 2021, Elsevier.

Researchers have made significant progress in animal studies related to orthopedic Zn‐based implants. Su et al. [127] implanted Zn‐0.5 V alloys into the aorta and femur, achieving a bone‐implant contact (BIC) rate higher than pure Zn, reaching up to 75%. Shao [55] used beagle dogs as the animal model and implanted Zn‐Mg‐Fe alloys into their frontal bone, mandible, and femur, observing them for one year. Through multidimensional analyses such as hematology, histology, and immunology, the researchers found that the alloy did not cause any abnormalities in blood, liver, kidney, or immune system. The Zn content in adjacent bone tissue increased in the short term and then decreased. The corrosion rate was approximately 0.183 mm/year after 3 months and decreased to about 0.065 mm/year after 12 months (Figure 25A) [55]. No significant differences in degradation were observed at different implant sites, indicating good biocompatibility and corrosion resistance. The degradation products were ZnO, Zn(OH)_2_, smithsonite, and Zn phosphate, and they were gradually replaced by new bone (Figure 25B) [55]. Jia [222] performed in *vitro* and in *vivo* experiments on pure Ti and Zn‐0.8Sr alloys, implanting them into the femoral condyles of rats for bone defect repair. After 12 weeks, Micro‐CT showed a 40% higher new bone volume fraction (BV/TV) in the Zn‐0.8Sr group compared to the pure titanium group (Figure 25C) [222]. The scaffold gradually degraded without causing inflammation, and the degradation products were well‐tolerated.

While the above orthopedic studies collectively report favorable biocompatibility, a critical comparison with vascular implantation data reveals marked site‐dependent inconsistencies in Zn degradation behavior. Drelich et al. [42] monitored pure Zn wires in the murine abdominal aorta for up to 20 months and reported a remarkably steady corrosion rate of approximately 0.02 mm/year with relatively uniform degradation morphology. Bowen et al. [22] similarly observed that pure Zn wires retained over 70% of their cross sectional area after 4 months in rat aortas. In contrast, the bone implantation data reviewed above show considerably different degradation kinetics: the Zn‐Mg‐Fe alloy in beagle bone exhibited an initial corrosion rate of 0.183 mm/year that decreased to 0.065 mm/year over 12 months [55] while Zn‐Li wires in rat aortas showed substantially different degradation patterns at comparable time points [223]. These discrepancies cannot be attributed solely to compositional differences between alloys; rather, they reflect the fundamentally different physicochemical environments at each anatomical site. In the vasculature, continuous blood flow provides stable pH buffering, efficient removal of corrosion products, and sustained oxygen supply, all of which favor relatively uniform surface dissolution [16, 32]. In osseous environments, however, the confined intramedullary or peri‐implant space limits electrolyte exchange, allows local accumulation of degradation products, and exposes the implant to a transiently acidic pH during inflammatory and bone‐remodeling phases [71, 73]. Subcutaneous tissue introduces yet another scenario, in which rapid fibrous encapsulation further restricts ionic transport [73, 79]. Notably, Shao et al. [55] reported no significant differences in degradation among three bone sites (frontal bone, mandible, and femur), suggesting that within a similar tissue class the local microenvironment may be sufficiently comparable.

### 5.3 Clinical Trials

For implants, the ultimate goal is to apply them to the human body. After animal studies have become relatively mature, clinical trials in humans are required. Currently, several studies have used models such as rats, rabbits, and dogs to verify the safety and efficacy of Zn‐based screws, plates, scaffolds, and other implants, but clinical application is still in the early exploratory stage. This stands in stark contrast to the clinical landscape for Mg‐based biodegradable implants, which have accumulated substantially more clinical evidence and regulatory approvals to date. Bone screws made of Mg‐Y‐RE‐Zr [224] and Mg‐Ca‐Zn [225] alloys have been approved by CE and the Korea Food and Drug Administration (KFDA) in 2013 and 2015, respectively. MAGNEZIX compression screws were evaluated in five studies including three RCTs comparing them with titanium screws [226]; the first RCT (*n* = 14) reported radiologic metallic debris and corresponding MRI artifacts in 3 patients, and the second RCT (*n* = 26) reported superficial wound‐healing problems in 3 patients up to 6 months, while a nonrandomized comparative study (*n* = 48) reported that degradation of Mg screws was completed by 12 months. More critically, MAGNEZIX pins degraded significantly faster and diminished to 77.54% ± 13.59% of the original implant area after only 16 weeks, and harmful hydrogen gas pockets were found, which correlated with reduced bone formation and a lower osteoblast count at 4 weeks [227]. In contrast, Zn‐based implants do not generate hydrogen gas during degradation, offering a meaningful clinical advantage in this respect.

**FIGURE 26:**
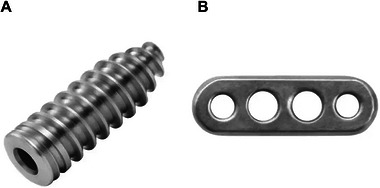
Clinical‐stage Zn‐based biodegradable devices. (A) Biodegradable interfacial screws and (B) zinc plates with loops developed by the Shangning Kezhi team of China. (Figure *source*: https://mp.weixin.qq.com/s/cKm049Mh8_JJLxL12VAigg).

Compared with Mg alloy implants, which have already achieved clinical application (e.g., the Magmaris [228] resorbable magnesium scaffold by Biotronik, Germany; the MAGNEZIX magnesium screw [229] by Syntellix AG, Germany), the clinical translation of Zn alloy implants remains in its infancy, with only a few Chinese companies having initiated human clinical trials to date. On June 8, 2023, the world's first clinical trial of a Zn alloy degradable interference screw and a looped Zn plate for anterior cruciate ligament reconstruction successfully enrolled subjects at Peking University Shougang Hospital, China (Figure 26). These devices, developed by Shangning Kezhi (Beijing, China), are based on a Zn‐Mn binary and ternary alloy system. Preliminary results show good safety and controllable degradation, with long‐term follow‐up and large‐sample data still being collected. More recently, on January 6, 2026, Suzhou Yuezhong Biotechnology Co., Ltd., China, in collaboration with Shanghai Jiao Tong University, China, announced that the world's first fully degradable Zn alloy surgical stapler had entered clinical trials at Xinhua Hospital Affiliated to Shanghai Jiao Tong University School of Medicine, China. The Zn alloy wire used in the stapler, designated JDBZ (Jiaoda Bio‐Zinc), reportedly achieves a record‐breaking tensile strength of 630 MPa while maintaining an elongation at break exceeding 40%, with an in *vitro* degradation rate of ∼250 µm/year and good biocompatibility, fully meeting the mechanical, degradation, and processing requirements for surgical staples.

### 5.4 Manufacturing and Scale‐Up

The transition of Zn‐based implants from laboratory‐scale prototypes to industrial‐grade medical devices necessitates overcoming significant metallurgical and engineering hurdles. The primary challenge in scaling production lies in the stringent control of impurity levels. Unlike traditional industrial Zn, biodegradable applications require ultra‐high purity (typically >99.99%) to prevent detrimental galvanic corrosion triggered by trace elements such as Fe, Ni, and Cu, which have extremely low solid solubility in Zn [71, 230].

Furthermore, ensuring microstructural homogeneity in large‐scale casting remains a technical bottleneck. As ingot sizes increase, macro‐segregation of alloying elements becomes more pronounced due to differences in density and cooling rates [231]. This chemical heterogeneity can result in inconsistent mechanical properties across the final product. To mitigate this, advanced thermomechanical processing (TMP) strategies, such as high‐ratio multi‐pass extrusion [232] or ECAP [233], must be optimized for industrial scales to ensure uniform grain refinement via dynamic recrystallization.

Additive manufacturing (AM) offers unparalleled potential for customized porous scaffolds; however, the high vapor pressure of Zn (6.1 × 10^2^ Pa at its melting point) poses a unique challenge [234]. During laser processing, the low boiling point of Zn (907°C) often leads to significant evaporation and “spatter” formation, resulting in internal porosity and compositional shifts [235]. Future industrial scaling will require specialized AM systems with pressurized chambers or optimized scan strategies to maintain the structural integrity and chemical precision of the printed implants.

### 5.5 Sterilization and Surface Effects

Sterilization is a non‐negotiable prerequisite for any implantable medical device [236]. The methods currently used for sterilization of implantable biomaterials and medical devices include autoclaving or steam sterilization, ethylene oxide sterilization, and sterilization using radiation emitted from various isotope sources [237, 238]. An important consideration for successful sterilization of implants is the selection of an appropriate sterilization method; first and foremost, the integrity of the implant biomaterials and medical devices must be maintained during and following the sterilization process [239]. Sterilization is an essential step in handling Zn‐based implants before their use in clinical practice, and various sterilization methods are available. Yet how these treatments influence Zn‐based biomaterials remains unknown and is of critical relevance.

Steam autoclaving, the most prevalent clinical sterilization technique, has proven incompatible with Zn based implants. Li et al. [240] evaluated three standard sterilization protocols, specifically gamma irradiation, hydrogen peroxide gas plasma, and steam autoclaving, on pure Zn and a Zn‐3Cu weight percent alloy. These materials were scrutinized to compare the distinct effects of sterilization on surface topographies, transient and long term degradation kinetics, and cellular toxicity. The results demonstrated that autoclaving brought about an apparently inhomogeneous zinc oxide film on the material surface, whereas the other two methods produced no apparent alterations on the material surfaces. Moreover, the samples after autoclaving showed significantly faster degradation rates and more severe localized corrosion, especially for the Zn‐Cu alloy. The steam autoclaving fundamentally alters the degradation profile and biological safety of Zn‐based implants prior to implantation, potentially invalidating pre‐clinical biocompatibility data and introducing unpredictable clinical risks.

Among the diverse modalities available for medical device sterilization, including moist heat, dry heat, radiation, and gaseous agents, the selection of an appropriate protocol for Zn‐based biodegradable implants is paramount [241]. Current experimental evidence identifies gamma irradiation and hydrogen peroxide gas plasma as the recommended modalities, as they exert no perceptible adverse effects on the biodegradability and cytocompatibility of Zn and Zn‐Cu alloys. In particular, gamma irradiation is highly suitable because it operates without elevating temperatures, thereby preserving the initial morphological characteristics, surface roughness, and wettability conditions of the specimen.

The transition of surface modified Zn‐based implants into surgical practice is significantly obstructed by the delicate nature of multilayered architectures, such as polydopamine or drug eluting films, which are frequently vitiated by conventional sterilization protocols [206, 242]. Ensuring that these nanostructured interfaces maintain their biochemical and structural integrity post sterilization is essential for fulfilling their intended therapeutic trajectories, yet the divergent responses of varied coating chemistries to gamma irradiation or ethylene oxide remain insufficiently characterized [243, 244]. Consequently, establishing standardized protocols is a critical requirement for the preclinical phase to ensure that in *vitro* and in *vivo* biocompatibility assessments accurately reflect the final implant state. Reproducing these complex multistep surface modifications on patient specific geometries at an industrial manufacturing scale further exacerbates the technical challenge, as the interplay between the initial surface topography and sterilization induced alterations remains a pivotal but rarely addressed hurdle in current literature.

### 5.6 Regulation Considerations

**FIGURE 27:**
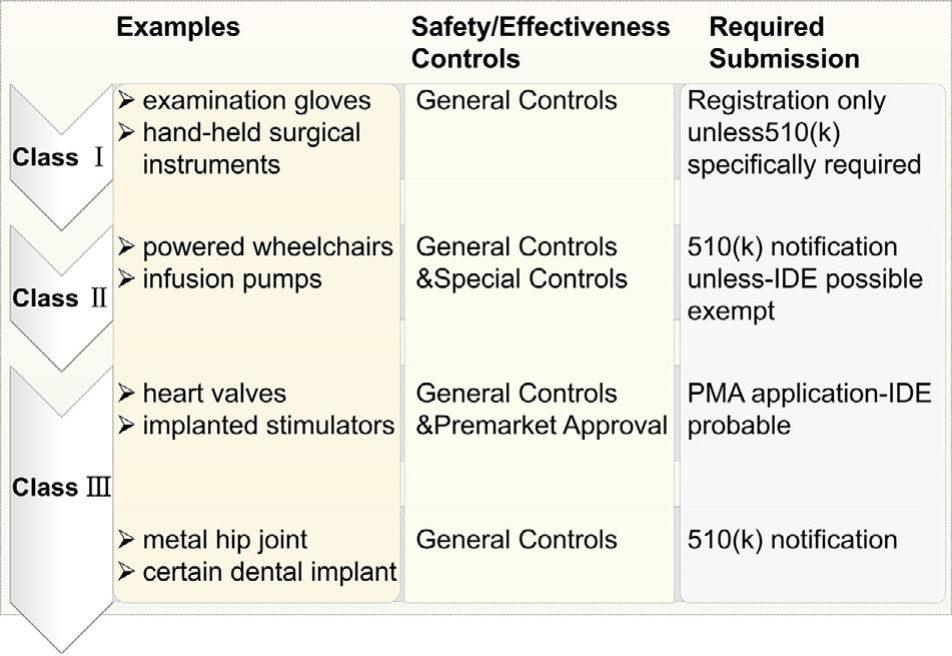
FDA medical device classification [248].

**FIGURE 28:**
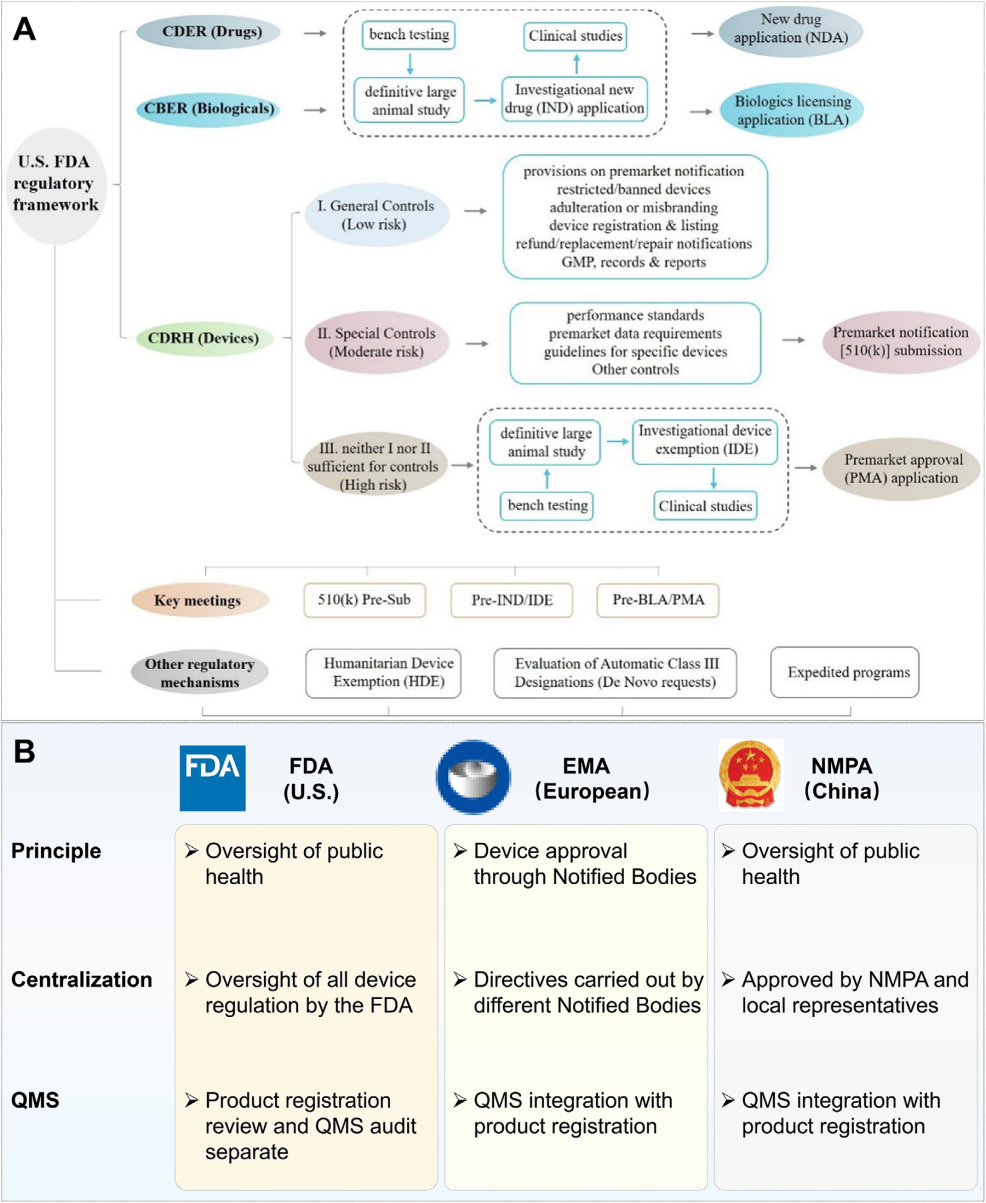
(A) The U.S. FDA regulatory framework [247]. Copyright 2023, Elsevier. (B) Comparison among FDA, EMA and NMPA for medical devices regulation [254].

The development and research of medical devices must comply with the standardized processes and regulatory review requirements set by regulatory agencies such as the FDA. Figure 28A illustrates the FDA's regulatory framework. The FDA classifies medical devices into three categories based on the risk they pose to patients (Figure 27). Orthopedic biodegradable Zn‐based implants fall under Class III [245]. During the product approval process, applicants are required to provide comprehensive safety and efficacy data [245, 246]. For Zn‐based biodegradable implants, it is necessary to provide a detailed explanation of the material composition, preparation process, degradation mechanisms, in *vitro* and in *vivo* performance test results, as well as preclinical animal studies and clinical trial data [247]. In addition to the FDA, regulatory agencies in other countries and regions have also introduced corresponding measures.

The European Union regulates medical devices through the CE certification system, which requires compliance with the EU Medical Device Regulation (MDR 2017/745). To obtain CE certification, products must undergo evaluation by a notified body, demonstrating that they meet the essential safety and performance requirements. Key areas of focus include risk management reports, the adequacy of clinical evidence, and post‐market surveillance plans. For biodegradable Zn‐based implants, compliance with relevant European standards is also necessary, such as the EN ISO 10993 series (biological evaluation of medical devices), among others [249, 250, 251, 252].

In China, the National Medical Products Administration (NMPA) is responsible for regulating medical devices. Devices are divided into three classes, and implantable products are placed in Class III, which has the most rigorous control. Approval involves several steps, including product testing, clinical trial permission, and a final registration review. These procedures aim to ensure stable product quality and to confirm that devices are safe and effective for clinical use [253]. Researchers have also compared the regulatory systems of the FDA (U.S. Food and Drug Administration), EMA (European Medicines Agency), and the NMPA, as summarized in Figure 28B.

Whether it's the FDA, EMA, or NMPA, these regulatory agencies, while operating independently in different regions, share common goals and work within unified international standards (ICH). They have established a strong global regulatory network through close regulatory cooperation and information sharing. These agencies enhance international collaboration and exchange to discuss and establish international standards and regulations for medical devices, promoting the global circulation and application of products in this field. By ensuring that drugs and medical devices within their jurisdictions are safe, effective, and of controlled quality, these agencies protect public health.

## 6 Limitations and Outlook

Over the past decade, substantial progress has been achieved in the development of biodegradable Zn‐based implants, particularly through alloying, microstructure tailoring and advanced surface engineering strategies to enhance mechanical integrity, corrosion behavior and biological performance. Although many optimized Zn alloys have demonstrated favorable outcomes in *vitro* and in preclinical animal models, their clinical translation remains at an early stage. To fully realize their clinical application potential, large‐scale, high‐quality clinical trials are urgently needed to evaluate long‐term safety, degradation kinetics, ion metabolism and functional outcomes across diverse patient populations and clinical scenarios.

A major challenge lies in the insufficient understanding of the multiscale mechanisms that govern Zn alloy degradation and the associated biological responses. While corrosion control and short‐term biocompatibility have been widely studied, the interactions among immune regulation, angiogenesis, osteogenesis and ion signalling in *vivo* remain poorly defined, limiting the rational design of next‐generation implants. To address this gap, the highest‐priority basic research direction is to establish a quantitative, multiscale theoretical framework that links the intrinsic material properties (alloy composition, microstructure, surface characteristics) to in vivo degradation behavior and downstream biological responses, with a specific focus on decoding the dose‐ and time‐dependent effects of Zn ion release on the immune‐vascular‐osteogenic cascade during tissue regeneration. Future efforts should focus on controlling the coupled effects of implant design, corrosion rate and the biological environment to ensure safe and reliable clinical use.

From a broader translational perspective, the long‐term systemic effects of Zn‐based implants remain insufficiently understood. Although Zn is an essential trace element, its metabolism is tightly regulated, and chronic elevation of systemic Zn^2+^ levels or accumulation of alloying elements (e.g., Li, Mn, Cu) requires careful examination. Long‐term tracking (≥12 months) of the biodistribution and clearance pathways of Zn^2+^ and alloying element ions in major organs (liver, kidney, and brain), together with discrimination of implant‐derived Zn from endogenous pools, is necessary. Moreover, the safety and behavior of implants in special populations, such as patients with impaired renal or hepatic function, systemic inflammation or osteoporosis, remain underexplored. In addition, regulatory standards for biodegradable metals are still developing, creating challenges for translation and commercialization.

In addition to the above challenges, several emerging frontiers warrant particular attention: (1) application of high‐throughput combinatorial experimentation and machine learning to accelerate alloy discovery beyond the current trial‐and‐error paradigm [7, 15, 244]; (2) integration with temporally programmed drug delivery systems to achieve multifunctional implants combining structural support, infection control and tissue regeneration [255, 256, 257]; (3) development of non‐invasive real‐time degradation monitoring technologies, such as biodegradable implantable sensors, to guide clinical decision‐making [258, 259, 260]; and (4) expansion into underexplored application domains including soft‐tissue repair and transient neural interfaces, where the anti‐inflammatory and antibacterial properties of Zn alloys may offer distinct advantages [261, 262]. Progress will require close cooperation among materials scientists, clinicians, industry and regulatory bodies to set clear rules for safety testing, long‐term monitoring and post‐market evaluation.

**FIGURE 29:**
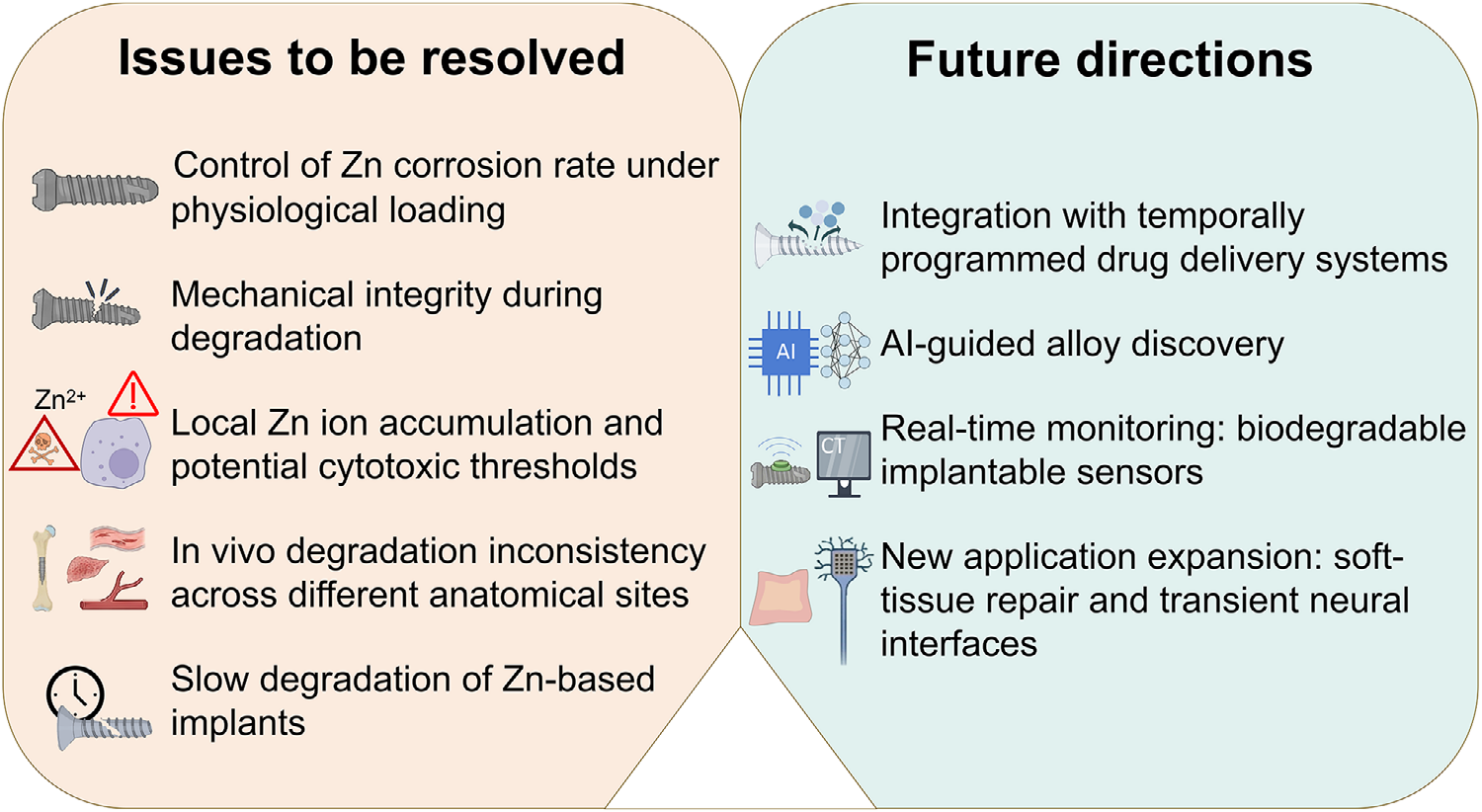
Issues to be resolved and future directions of biodegradable Zn‐based implants.

Overall, Zn‐based biodegradable implants hold significant promise for orthopedic, cardiovascular and soft‐tissue repair applications due to their favorable biocompatibility, biological functionality and tunable degradation behavior. Future progress will require a concerted effort to deepen mechanistic understanding, standardize evaluation systems, harness advanced manufacturing technologies and accelerate clinical validation. With these advances, Zn‐based implants are poised to become an important new class of next‐generation bioresorbable medical devices (Figure 29).

## 7 Conclusions

Biodegradable Zn‐based implants have emerged as a highly promising class of bioresorbable materials owing to their balanced degradation behavior, favorable biocompatibility, and multifunctional biological activities. This review discussed recent advances in Zn‐based implant systems, including alloying strategies, surface modification approaches, antibacterial regulation, degradation evaluation methods, and preclinical/clinical studies, together with the current status of relevant regulatory frameworks.

Although significant progress has been achieved in improving the mechanical properties, corrosion behavior, and biological performance of Zn‐based materials, several critical issues remain unresolved before widespread clinical application can be realized. In particular, a deeper understanding of the relationships among alloy composition, degradation behavior, ion release, and host biological responses is still required. In parallel, the establishment of standardized evaluation systems, long‐term biosafety assessments, and clinically relevant validation protocols will be essential for future translation.

Overall, with continued advances in mechanistic understanding, intelligent material design, advanced manufacturing technologies, and clinical validation, biodegradable Zn‐based implants are expected to play an increasingly important role in orthopedic, cardiovascular, and soft‐tissue repair applications, thereby accelerating the development of next‐generation bioresorbable medical devices.

## Author Contributions

**Panfeng Zhao**: writing – original draft, formal analysis, data curation, conceptualization. **Zhengquan Wang**: writing – review & editing, supervision, resources, project administration, funding acquisition. **Hui Yang**, **Xiyuan Zhang**, **Hanyuan Liu**, and **Xi Zhao**: data curation. **Yanyan Liu**, **Xiaotong Lu**, and **Xiaocheng Li**: conceptualization. **Wenhao Zhou**: conceptualization, data curation, funding acquisition. **Yufeng Zheng**: resources, conceptualization, data curation.

## Conflicts of Interest

The authors declare no conflicts of interest.

## Data Availability

Data sharing not applicable to this article as no datasets were generated or analysed during the current study.
